# Homo and Heterotypic Cellular Cross-Talk in Epithelial Ovarian Cancer Impart Pro-Tumorigenic Properties through Differential Activation of the Notch3 Pathway

**DOI:** 10.3390/cancers14143365

**Published:** 2022-07-11

**Authors:** Souvik Mukherjee, Asmita Sakpal, Megha Mehrotra, Pratham Phadte, Bharat Rekhi, Pritha Ray

**Affiliations:** 1 Imaging Cell Signaling and Therapeutics Lab, Advanced Centre for Training Research and Education in Cancer, Navi Mumbai 410210, India; smukherjee@actrec.gov.in (S.M.); lgunjal@ymail.com (A.S.); meghameh13@gmail.com (M.M.); pratham1122@gmail.com (P.P.); 2 Homi Bhabha National Institute, BARC Training School Complex, Anushaktinagar, Mumbai 400094, India; rekhi.bharat@gmail.com; 3 Tata Memorial Hospital, Dr. E Borges Road, Parel, Mumbai 400012, India

**Keywords:** Notch3 signaling, co-culture, epithelial ovarian cancer, imaging

## Abstract

**Simple Summary:**

The omental metastatic spreading of epithelial ovarian cancer is spearheaded by complex cell–cell interactions present in the fluidic microenvironment (ascites), which is yet to be fully decoded. Using a unique co-culture model of SNFT (SKOV3 cells expressing a Notch3 luciferase reporter-sensor) and NIH3T3 cells (differentially overexpressing Jagged1 ligand), we demonstrated that incremental Jagged1 expression led to proportional Notch3 activation in SNFT. Differential Notch3 activation was also evident from co-culture of SNFT with other EOC cell lines and ascites-derived cancer-associated fibroblasts of HGSOC patients expressing varying levels of Jagged1. Amongst the top five modulated genes identified by the gene profiler array, both p21 and VEGFA showed enhanced expression (for p21)/secretion (for VEGFA) in SNFT when induced with Jagged1. Secreted VEGFA further reduced CSC differentiation in platinum-resistant A2780 cells. Pronounced VEGFA expression associated with Notch3 up-regulation in metastatic HGSOC tumors delineates an unknown role of the Notch3/VEGFA axis in EOC progression.

**Abstract:**

An active fluidic microenvironment governs peritoneal metastasis in epithelial ovarian cancer (EOC), but its critical functional/molecular cues are not fully understood. Utilizing co-culture models of NIH3T3 cells (differentially overexpressing Jagged1) and SKOV3 cells expressing a Notch3 luciferase reporter-sensor (SNFT), we showed that incremental expression of Jagged1 led to proportional Notch3 activation in SNFT. With no basal luciferase activity, this system efficiently recorded dose-dependent Notch3 activation by rh-Jag1 peptide and the non-appearance of such induction in co-culture with NIH3T3^Δjag1^ cells indicates its sensitivity and specificity. Similar Notch3 modulation was shown for the first time in co-cultures with HGSOC patients’ ascites-derived cancer-associated fibroblasts and Jagged1-expressing EOC cell lines. NIH3T3^J1-A^ and OVCAR3 co-cultured SNFT cells showed maximum proliferation, invasion, and cisplatin resistance among all the heterotypic/homotypic cellular partners. VEGFA and CDKN1A are the two most upregulated genes identified across co-cultures by the gene profiler array. Co-culture induced VEGFA secretion from SNFT cells which also reduced cancer stem cell differentiation in platinum-resistant A2780 cells. rh-Jag1-peptide promoted enhanced nuclear-cytoplasmic p21 expression. Additionally, metastatic HGSOC tumors had higher VEGFA than corresponding primary tumors. This study thus demonstrates the tumoral and non-tumoral cell-mediated differential Notch3 activation imparting its tumorigenic effects through two critical molecular regulators, VEGFA and p21, during EOC progression.

## 1. Introduction

Cell-to-cell communication is an important way of fostering a pathogenic microenvironment. The reciprocation among cancer cells and the remaining cellular/acellular components in the tumor microenvironment leverages the disease and makes clinical intervention challenging. The microenvironment comprises cancer cells, cancer-associated fibroblasts (CAFs), endothelial cells, mesenchymal stem/stromal cells (MSCs), pericytes, immune cells, and cell-derived components such as ascites, cytokines, metabolites, extra-cellular vesicles, etc. [1]. This milieu provides several interactions ranging from classical para/endocrine cues to transient cross-talks like phagocytosis, pinocytosis, receptor-ligand, and many others. These interactions are both context- and tissue- dependent and influence crucial tumorigenic functions, such as immune-suppression, chemoresistance, angiogenesis, and metastasis, that often rely upon the levels of interactors (e.g., molecules/cells) [2]. Assessment of the intra- and inter-cellular milieu is thus critical to understanding the course of the disease and biological consequences that pave the way to developing therapeutic strategies.

Unlike other solid tumors, Epithelial ovarian cancer (EOC) primarily spreads through direct peritoneal dissemination known as transcoelomic metastasis. Cell-to-cell interactions in the tissue and fluidic (ascites) microenvironment are crucial in the early stages of progression, and the omentum acts as the initial attachment site, followed by the visceral-parietal peritoneum [3]. The omental vasculature displays branches of blood vessels ending in a glomerulus-like capillary bed alongside several immune cells (T-cells, NK-cells, macrophages, etc.) aggregating in/around them below the adipocytes. This site is known as the milky spot, the hotbed for peritoneal metastasis [4]. A syngeneic murine ovarian cancer model has shown that, post intraperitoneal injection of ID8 cells, metastasis happened within a week in the omentum, which remained the only site of metastasis for six weeks [5]. Nieman et al. highlighted that the adipokines and fatty acids secreted from the milky spot that promote omental metastasis in EOC [6]. However, the molecular factor/s arising from cell–cell interactions, instrumental in promoting the disease, are largely unknown.

Despite widespread peritoneal metastasis, the underlying mechanisms, non-random distribution, and cell implantation have not been completely deciphered. Techniques like SNP array, karyotyping, and transcriptomics identified Notch3, a single-pass transmembrane receptor heterodimer, as altering (mostly as gene amplification and increase in mRNA copies) 21% of high grade serous ovarian carcinomas (HGSOC), while 61% of all patients have at least one alteration in the Notch3 pathway. Among the remaining receptor isoforms, the alterations are 9% for Notch1 and Notch4 and 14% for Notch2 [7,8]. Interestingly, unlike Notch3, patients harbored mis-sense mutations and deletions in the remaining Notch receptors. Although Jagged1 is the third-most altered (7%) ligand of the Notch pathway after DLL3 (15%) and Jagged2 (8%) in the tumor, previous studies have identified that the omental mesothelial cells predominantly express Jagged1 [9]. However, measuring such interactions in real-time to assess the subsequent outcome is indispensable to determining the course of the disease yet not attempted.

In this study, a unique homotypic/heterotypic co-culture system comprising a Notch-reporter sensor expressing SKOV3 (SNFT), other EOC cell lines (homotypic), and NIH3T3/patient-derived fibroblasts (heterotypic) demonstrated that Notch3 signaling activation is critically dependent upon the threshold level of ligand (Jagged1) expression in real-time. Differential levels of Jagged1 impart differential cleavage of the Notch3 receptor, leading to proportional augmentation of proliferation, chemoresistance, and invasion abilities in the tumor cells. Intriguingly, using a Notch3 pathway gene profiler array, two atypical Notch3 targets, cyclin-dependent kinase inhibitor 1A (CDKN1A) and vascular endothelial growth factor A (VEGFA), were identified as the most upregulated factors across both the homotypic and heterotypic interactions. Functional validation of these novel targets highlights the robustness of our system and enhances knowledge of the molecular cues that guide the EOC metastasis.

## 2. Material and Methods

### 2.1. Reagents and Antibodies

Cisplatin (P-4394), α-tubulin (T5168), anti-Notch3 (HPA044392—that exclusively detects the Valine-1744 residue of NICD3 (notch intracellular domain3) fragment), anti-mouse (12–349), and anti-rabbit (A6154) HRP tagged, Verapamil (V4629) and Gamma-secretase inhibitor ((GSI: N-[N-(3,5-Difluorophenacetyl)-L-alanyl]-S-phenylglycine t-butyl ester (DAPT)) (D5942) were purchased from Sigma-Aldrich (St. Louis, MW, USA). IHC detection kit (ab236466), Jagged1 (ab77751) and Hes1 (ab71559) antibodies were from Abcam (Cambridge, UK). Pbx1/2 (sc-28313) and p21 (2947S) antibodies were from Santa-Cruz (sc-28313) and Cell Signaling Technology. The 17-mer rh-Jag1 peptide (CDDYYYGFGCNKFCRPR) (188–204), corresponding to amino acids 187–203 of human Jagged1 peptide, was purchased from Ana spec (Fermont, CA, USA). Dye cycle violet (DCV) was purchased from Invitrogen (Waltham, MA, USA).

### 2.2. Cell Lines & Notch3 Reporter Sensor

Mouse embryonic fibroblast line NIH3T3 and EOC cell line A2780 were cultured in DMEM. Other EOC cell lines, e.g., SKOV3, TOV21G, OVCAR3, and OAW42, were cultured in RPMI and MEM, respectively. All media were supplemented with 10% FBS (20% for OVCAR3) and 1% penicillin-streptomycin. Neomycin selection was used to express Jag1 in NIH3T3 stably. Differential Jagged1 expressing clones (NIH3T3^jag1^) were selected based on MFI values and membrane localization phenotype. A2780^LR^ cells were generated earlier as a model for acquired chemoresistance by the dose-escalation method using cisplatin [10].

10X-CSL (CBF-Su(H)-Lag2), a promoter response element, comprising 10 NICD response element CSL-binding sites (5′-GTGGGAA-3′), was cloned upstream of a bifusion reporter (firefly luciferase fused with tandem Tomato red fluorescence protein or *fl2-tdT*) [11]. SKOV3 cells (Notch3^+^/NICD3^−^), stably expressing the construct, were selected by neomycin.

### 2.3. Co-Culture Assay

The NIH3T3, NIH3T3^jag1^, other cancer cells, and patient-derived CAF were used as a feeder layer for SNFT co-culture. All the feeder cells were seeded a day prior and treated with mitomycin C (Mito-40, Neon) (4 μg/mL for 2 h). On day 0, SNFT cells were overlaid in a specific ratio, and co-culture was performed as per experimental requirements. DAPT inhibition was given for 48 h, and the concentration was decided as per the experimental requirements.

### 2.4. Immunofluorescence and Western Blotting

The cells were fixed using 4% paraformaldehyde, permeabilized with 0.2% Triton-X-100) followed by blocking with 3% BSA, and sequentially probed with a primary antibody (at 4 °C overnight) followed by a secondary antibody (2 h at room temperature). DAPI was used for counterstaining, and the coverslips were mounted using Vectashield (H-1500, Vector laboratories, Newark, CA, USA). Immunofluorescence images were acquired using Carl Zeiss, LSM 780 microscope, and processed by ImageJ (version: 1.8.0_172). For the p21 localization study, we used the Intensity Ratio Nuclei Cytoplasm Tool.

As described earlier, whole-cell lysates preparation and Western blotting were performed [10]. The blots were developed in chemidoc (Bio-Rad, Hercules, CA, USA), and the image lab software performed the densitometry.

### 2.5. Luciferase Assay and Imaging

Luciferase assay to evaluate the activity of Notch response elements was performed using a Promega Luciferase assay system (E1500) (Madison, WI, USA) by Cytation 5 cell imaging multi-mode reader. For bioluminescence imaging, 200 µg of D-luciferin was added to each well, imaged in Xenogen-IVIS, and quantified by Living Image software 4.4.

### 2.6. Flow Cytometry

Sorting of side and non-side population cells was performed as described in a prior study [12]. Verapamil (50 µM), a drug transporter inhibitor, was used as a negative control for gating. For determination of cell surface expression of Jag1, cells were blocked in FACS buffer followed by staining with primary and secondary antibodies. In the case of Oct4 staining, additionally, cells were fixed with 2% PFA and permeabilized with 0.2% triton-X-100.

CFSE dye dilution assay was performed to determine SNFT proliferation after co-culture. SNFT cells, synchronized by mimosine treatment for 16 h, were stained with 10 µM Carboxyfluorescein succinimidyl ester (CFSE) (34554, Thermo Scientific, Waltham, MA, USA) as per the manufacturer’s protocol. The cells were then equally divided, and one part was acquired to determine the viable cells by FACS ARIA III using FACS DIVA software. At least 20,000 events were acquired with propidium iodide for gating the viable cells in FACS ARIA III using FACS DIVA software. The remaining cells were co-cultured with different feeder layers of NIH3T3 or NIH3T3^jag1^ cells already treated with mitomycin C in a 1:2 to 1:3 ratio depending upon the cell size. The cell proliferation was monitored for 48 h, and the mean fluorescence intensity (MFI) was calculated using FlowJo VX. The proliferation index (PI), a ratio-metric value of the total number of generations and number of cells having undergone any number of divisions, was derived from the MFI using the following equation.

$$PI=\frac{\sum_{1}^{i}i\times \frac{N_{i}}{2i}}{\Sigma_{1}^{i}\frac{n_{i}}{2^{i}}}$$

*N_i_* = total number of cells undergoing division till *i*th generation.

### 2.7. Cell Invasion Assay

A transwell invasion assay was carried out to assess the cellular invasion property. The IncuCyte ClearView inserts (Essen BioScience, Ann Arbor, MI, USA) were coated with Matrigel (50 μg/mL) prepared in the incomplete culture medium. CFSE-labelled SNFT cells were seeded either alone or with NIH3T3/NIH3T3^jag1^ clones/other EOC cell lines in the inserts and grown in a serum-free medium for 24 h. The bottom reservoir was filled with a complete medium. 1500 SNFT was co-cultured for 24 h in the ratio above, and the bottom plane of the insert was imaged by Incucyte live cell analysis instrument. The green puncta were counted by the Incucyte software (version-2020A).

### 2.8. Cell Cytotoxicity Assay

CFSE-labelled SNFT cells were co-cultured either with NIH3T3 or NIH3T3^jag1^ clones or EOC cell lines for 24 h, followed by segregation through flow cytometry. From the sorted population, 2000 cells were seeded and treated with different concentrations of cisplatin for 48 h. Cell viability was determined by 3-(4,5-dimethylthiazol-2-yl)-2,5-diphenyl tetrazolium bromide (MTT) (M2003, Merck, Burlington, MA, USA) assay.

### 2.9. VEGFA Enzyme-Linked Immunosorbent Assay (ELISA)

100 microliter of culture-media from SNFT (alone or NIH3T3^wt^/NIH3T3^jag1^ clones/EOC cell line co-culture) were used for the sandwich ELISA (E-EL-H0111, Elabscience, Houston, TX, USA) following the manufacturer’s protocol to determine the concentrations of secreted VEGF using a standard curve. The concentration of the standards ranged from 31.25 to 2000 pg/mL.

### 2.10. Notch3 Immunohistochemistry

Sections of the FFPE tumor blocks were made with 5-micron thickness. Following de-waxing, peroxidase activity was blocked, and the antigen was unmasked with pH 6 buffer by the HIER method. Post incubation with primary/secondary antibodies, the sections were developed by DAB, followed by haematoxylin counterstain, and mounted by DPX. The slides were graded blindly by an experienced pathologist, and the score was represented by the Immunoreactivity index.

### 2.11. RNA Isolation

For isolation RNA from SNFT cells post-co-culture, different feeder cells were labelled with CFSE followed by their cell-division arrest, as mentioned earlier, and then co-incubated with SNFT for 24 h. The CFSE-negative population was collected and allowed to adhere first, and total RNA was extracted by Qiagen RNeasy mini kit (74104, Germantown, MD, USA).

### 2.12. Quantitative Real-Time PCR and Notch3 Gene Profiler Array

cDNA was synthesized from RNA extracted from EOC cell lines using a SuperScript IV First-Strand Synthesis kit (18090010, Invitrogen, Waltham, MA, USA). Quantitative real-time-PCR was performed using PowerUp SYBR Green Master Mix (A25741, Invitrogen) using appropriate gene-specific primers with Glyceraldehyde-3-phosphate-dehydrogenase (GAPDH) as an internal control. The relative gene expression was measured as delta Ct values. The list of primer sequences is given in the supplementary information (Table S1).

The real-time RT^2^ Profiler PCR Array (QIAGEN, Cat. no. CLAH40080E, Germantown, MD, USA) and RT^2^ SYBR^®^ Green qPCR Master Mix (330529) were used for transcriptional profiling. GAPDH was the assay reference gene. Ct values were derived to an Excel file to build a table and then uploaded onto the data analysis web portal at http://www.qiagen.com/geneglobe (accessed on 8 December 2021) (Table S3).

### 2.13. Isolation of Mesothelial Cells from Patients

Ascitic fluid enriched with tumor cells, CAFs, leucocytes, and other immune cells was collected from high-grade serous ovarian cancer patients through ascites tapping as per protocol approved by the Ethics Committee-III of ACTREC-TMC. Cells were sorted into CD90^+^ and CD90^−^ populations by MACS (Miltenyi Biotec GmbH, Bergirsch, Gladbach, Germany), and the CD90^+^ CAF fraction was further tested for the absence of EpCAM expression by immunofluorescence. Jagged1 expression was determined either by immunofluorescence/flow cytometry or both, and CAFs with membranous Jagged1 were further used for experiments.

### 2.14. Statistical Analysis

All the data represented the mean ± SEM of at least three independent experiments and were analyzed for significance using paired/unpaired Student’s *t*-test. *p*-value ≤ 0.05 was considered significant. For gene regulation calculations, we chose a ±2-fold cut-off for considering significantly altered candidate genes.

## 3. Results

### 3.1. Establishment of Differential NIH3T3^jag1^ Clones and Notch3 Reporter Sensor

**Figure 1:**
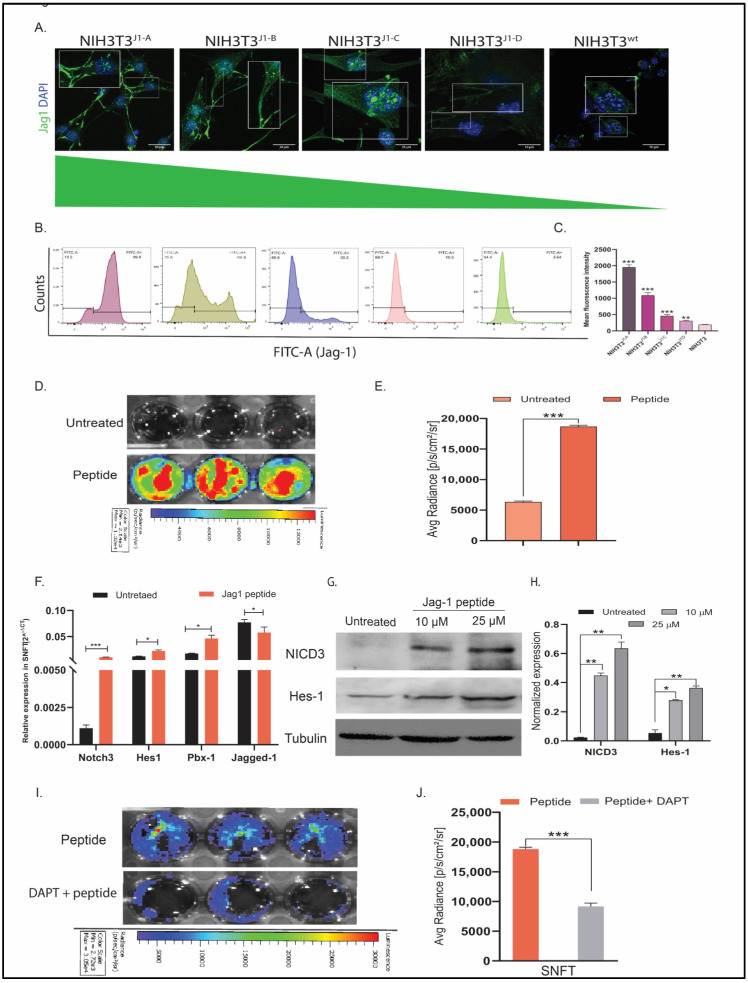
Establishment of differential NIH3T3^jag1^ clones and Notch3 reporter sensor. (**A**) Immunofluorescence images representing the membranous localization of Jag1 in NIH3T3 clones (white outlined inset images showed the magnified view of the areas highlighted). (**B**,**C**) Flow cytometry panel representing the quantitation of cell surface expression of Jag1 through mean fluorescence intensity, which indicated a differential degree of Jag1 expression across the clones (*n* = 3). (**D**,**E**) Live cell bioluminescence imaging of SNFT cells after Jag-1 peptide treatment (**D**) and graphical representation of the same (*n* = 2) (**E**) showing increased Notch3-sensor promoter activity. (**F**,**G**) Several Notch3 targets (hes1, pbx1, notch3) showed upregulation after peptide-induced activation at the transcript level (*n* = 3). Further, the Western blot of NICD3 and Hes1 after differential Jag1 induction in SNFT (**G**) (also see Figure S6A) and the densitometric quantification of the proteins (**H**) (housekeeping control: alpha-tubulin, *n* = 2) highlight that the NICD3 cleavage and its target protein expression increased differentially upon induction due to the pathway activation. (**I**,**J**) Activation/inhibition kinetics of live-cell bioluminescence image depicting that peptide-induced promoter activity attenuated after DAPT blockade (*n* = 2). * *p* ≤ 0.05, ** *p* ≤ 0.01, *** *p* ≤ 0.001.

For elucidating the differential activation of Notch3 by variable Jagged1 expression, NIH3T3 was stably expressed with different Jagged1 levels and validated by immunofluorescence and FACS. All the NIH3T3^jag1^ clones possessed prominent and patchy membranous Jagged1 (Figure 1A). The activation of Notch3 critically depends upon the ligand’s membrane localization. Flow cytometry analysis showed differential expression of Jagged1 in four clones, further selected for the study (NIH3T3^J1-A^, NIH3T3^J1-B^, NIH3T3^J1-C^, and NIH3T3^J1-D^ showed ~10-fold, ~6-fold, ~2.5-fold and ~1.5-fold-increase over NIH3T3^jag1wt^, respectively) (Figure 1B,C). The transcript and protein levels (data not shown) also validated the differential expression of Jagged1 across the clones, and mRNA/protein had a good positive correlation (r = 0.87) (Figure S1A).

The notch reporter sensor was stably expressed in SKOV3 (SNFT cells) for spatiotemporally assessing the consequence of Notch3 activation. Since the CSL domain provides a generalized measure of Notch receptor activation, the exact effect of Notch3 was investigated by probing with a NICD3-specific antibody. Moreover, SKOV3 possesses all cleaved-Notch isoforms but NICD3 [13]. Rh-Jag1 (10 µM) treated SNFT caused bioluminescence signal induction due to NICD3, which was ~2.9-fold higher than the untreated cells (Figure 1E). Untreated SNFT did not show any detectable luminescence, indicating that signal enhancement was the specific effect of NICD3. Rh-Jag1 also increased Notch3 target gene expression such as *hes1* (1.87-fold, *p* < 0.05), *pbx1* (2.64-fold, *p* < 0.05) (Figure 1F). Different peptide concentrations (10 µM and 25 µM) showed 19-fold (*p* < 0.001) and 27.4-fold (*p* < 0.001) higher NICD3 and 5.14-fold (*p* < 0.01) and 7.72-fold (*p* < 0.001) higher hes1 expression at the protein level in SNFT (*n* = 2) (Figure 1G,H). DAPT (10 µM) reduced the peptide-induced activation by 2.05-fold (*p* < 0.001) (Figure 1I,J).

### 3.2. Establishment of a Unique Co-Culture Model of Differential Notch3 Activation by SNFT and NIH3T3^Jag1^ Cells

**Figure 2:**
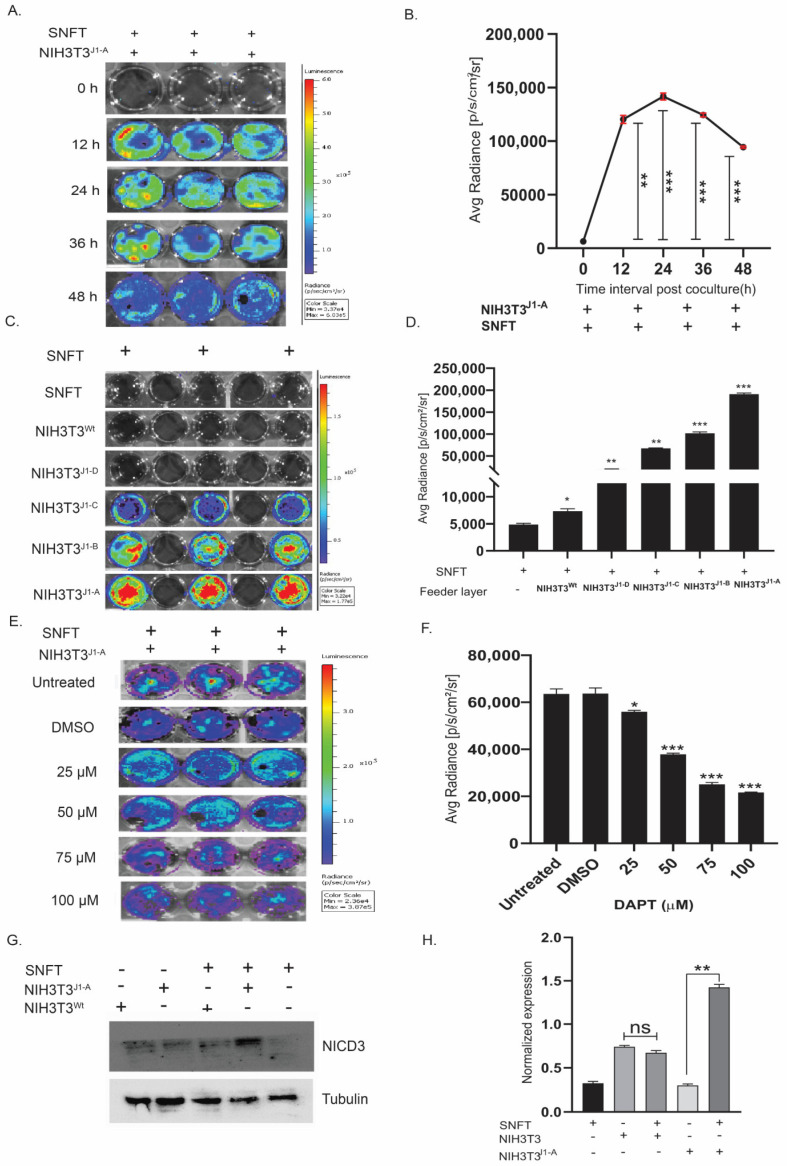
Establishment of a unique co-culture model of Notch3 activation-sensor expressing tumor cells (SNFT) and differentially expressing Jagged-1 fibroblasts (NIH3T3^jag−1^). (**A**,**B**) Temporal kinetics of Notch3 sensor activity in SNFT in co-culture with NIH3T3^J1-A^ cells showed an initial activation after 12 h of 18.9-fold (*p* < 0.01), which reaches its maxima at 24 h (22.3-fold, p < 0.001) and subsequently dropped by 48 h (14.8-fold, *p* < 0.001). The two-tailed error bar (red) represented the SEM (*n* = 3). (**C**,**D**) Bioluminescence images of co-culture between SNFT and different Jag1 clones of NIH3T3 (**C**) show a linearly proportional relationship between Jagged1 expression and Notch3 promoter activity as represented graphically in (**D**) (*n* = 3 for each clone). (**E**,**F**) Dose-inhibition kinetics are represented by the live-cell images (**E**) and graphically (**F**), which shows that concentration between 50 µM to 75 µM of DAPT results in a significant reduction in the NIH3T3^J1-A^ mediated Notch3 activity in SNFT (*n* = 3). All three real-time live-cell imaging experiments were independently performed and represented by respective scale bars. (**G**,**H**) Immunoblot of sorted SNFT cells post-co-culture and graphical analysis show increased release of NICD3 in SNFT when co-cultured with NIH3T3^wt^ (not significantly) and with NIH3T3^J1-A^ (~3-fold, *p* ≤ 0.01). The ‘+’ sign and ‘−‘ sign signify the presence and absence of the cells in co-culture, respectively (*n* = 2) (also see Figure S6B). * *p* ≤ 0.05, ** *p* ≤ 0.01, *** *p* ≤ 0.001.

We cultured the SNFT cells upon the NIH3T3^jag1^ clones (feeder layer) and measured Notch3 luminescence as a read-out of Notch3 activation. In co-culture, NIH3T3^J1A^-induced Notch3 activity in SNFT was first observed at 12 h, which increased by ~19-fold (*p* < 0.01) from the initial time point (6340 ± 157.16 p/s/cm^2^/sr), further enhanced at 24 h (22.3-fold, *p* < 0.001), and temporal declined at 36 h (19.6-fold, *p* < 0.001) and 48 h (15-fold, *p* < 0.001) (Figure 2A,B). Therefore, 24 h was selected for incubation for all experiments. Co-culture of SNFT with all the NIH3T3^jag1^ clones (NIH3T3^wt^, NIH3T3^J1-D^, NIH3T3^J1-C^, NIH3T3^J1-B^, NIH3T3^J1-A^) resulted in a linearly proportional increase in promoter activity by 1.5-fold, 3.9-fold, 13.8-fold, 20.9-fold, and 39.3-fold, respectively, compared to SNFT alone (Figure 2C,D). A dose-dependent decrease in Notch3 reporter activity level was observed with increasing DAPT concentration, and maximal inhibition (3.16-fold) was observed at 75 µM (for 48 h) (*p* < 0.001) (Figure 2E,F). No detectable luciferase activity was observed on co-culturing Jag1nod mutant expressing NIH3T3 cells with SNFT cells, suggesting the system’s specificity (Figure S1B–D).

Finally, NIH3T3^J1-A^, the highest *Jag1* expressing clone, enhanced NICD3-cleavage in SNFT compared with NIH3T3^wt^ co-culture suggesting the presence of an active juxtacrine cross-talk (Figure 2G,H).

### 3.3. Differential Induction of Notch3 by Homo/Heterotypic Cellular Interactions Leads to Differential Modulation in Proliferation, Invasiveness, and Cisplatin Sensitivity in SNFT

**Figure 3:**
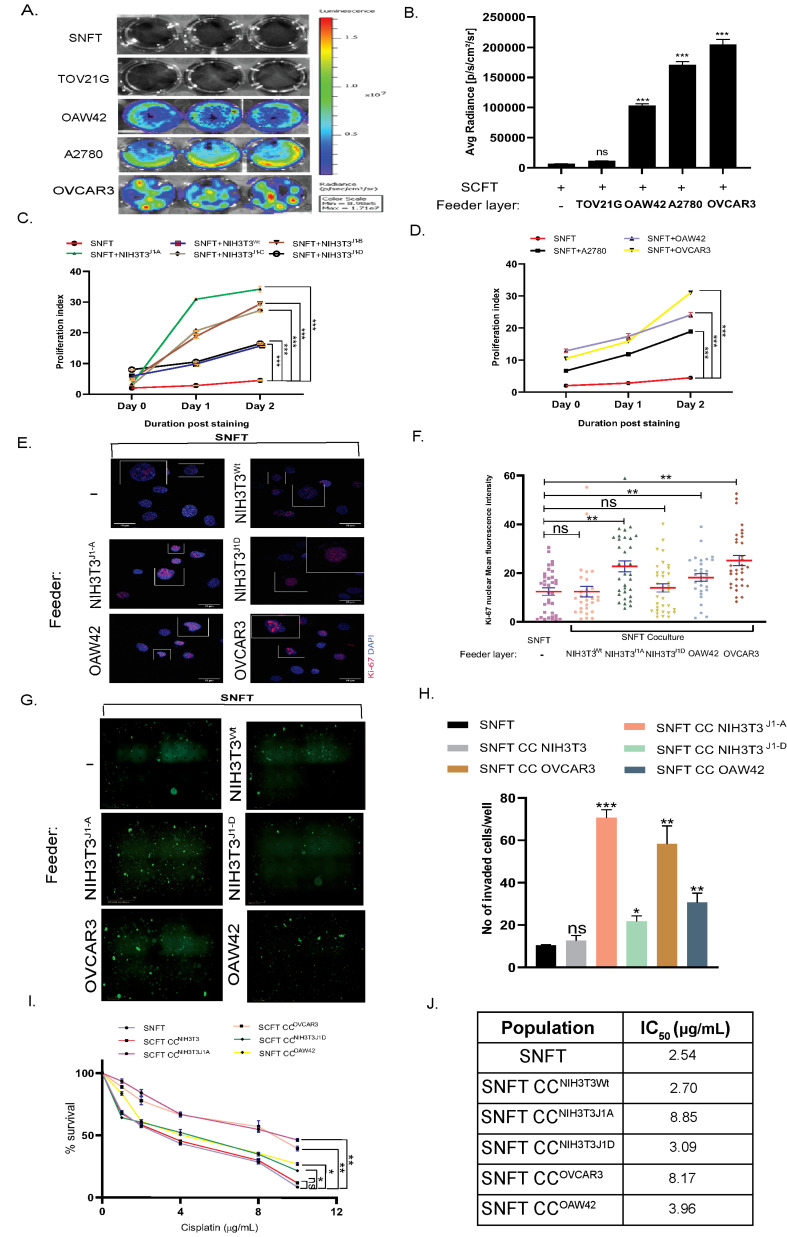
Differential induction of Notch3 by homo/heterotypic interactions leads to modulation in proliferation, invasiveness, and cisplatin sensitivity in SNFT. (**A**,**B**) Bioluminescence images of SNFT individually co-cultured with TOV21G, OAW42, A2780, and OVCAR3 cell lines naturally expressing different levels of Jag1, which led to differential Notch3 promoter activity as represented by the bar diagram (*n* = 3). Co-culturing with TOV21G showed comparable luciferase activity (9111 ± 458.28 p/s/cm^2^/sr) to SNFT. (**C**,**D**) The proliferation index was calculated by FlowJo software and plotted as a line graph which indicated heterogeneity in the proliferation rate of SNFT post-co-culture with different NIH3T3-Jagged1 expressing clones and EOC cell lines. The maximum increase in SNFT proliferation was found after NIH3T3^J1-A^ co-culture (12.3-fold, *p* < 0.001) and post OVCAR3 co-culture (7.7-fold, *p* < 0.001) on day2. (**E**,**F**) Immunofluorescence images of Ki-67 staining, a nuclear marker for cell proliferation in SNFT cells sorted after co-culturing with various EOC cells and Jagged-1 expressing NIH3T3 clones labelled with CFSE (**E**). Graphical comparison of the overall distribution of Ki-67 labelled SNFT cells exhibited maximal induction after co-culture with OVCAR3 cells, followed by NIH3T3^J1-A^ and OAW42 cells. NIH3T3^wt^ also induced Ki-67 expression SNFT but not significantly (*n* = 2). * *p* ≤ 0.05, ** *p* ≤ 0.01, *** *p* ≤ 0.001. (**G**,**H**) The epifluorescence images represent the invaded SNFT cells (green) after co-culture (**G**). The highest invasion was observed in SNFT co-cultured with NIH3T3^J1-A^, followed by OVCAR3, OAW42, and NIH3T3^J1-D^ (**H**) (*n* = 3). (**I**,**J**) The result of the MTT assay was plotted as drug concentration vs. percentage cell survival in a line graph which showed that across different co-culture conditions NIH3T3^J1-A^ and OVCAR3 imparted the highest cisplatin resistance in SNFT, which gradually decreased with OAW42, NIH3T3J1-D, and NIH3T3^Wt^ co-cultures. The IC_50_ doses of SNFT alone and after co-culture with different cells are presented in the table (*n* = 3). * *p* ≤ 0.05, ** *p* ≤ 0.01, *** *p* ≤ 0.001.

We confirmed the Jagged1 expression/membrane localization of EOC cell lines (e.g., A2780, OVCAR3, TOV21G, and OAW42) and utilized them as a feeder for juxtacrine-activation of Notch3 (Figure S2A–C). Upon co-culturing with SNFT, OVCAR3 induced the maximum activation (204,333.33 ± 8412.95 p/s/cm^2^/sr) followed by A2780 (170,666.66 ± 5456.90 p/s/cm^2^/sr) and OAW42 (97,349.27 ± 3247.22 p/s/cm^2^/sr) (*p* < 0.001) (Figure 3A,B). DAPT (50 µM) significantly attenuated this activity (A2780:1.68-fold, *p* < 0.001; OAW42:2.36-fold, *p* < 0.001; OVCAR3:1.89-fold, *p* < 0.01) (Figure S2D,E). Despite expression at the RNA and protein levels, TOV21G showed no membranous Jag1 (data not shown). Immunoblot showed NICD3 release in SNFT post-co-culture with OVCAR3 and OAW42 but not with TOV21G (Figure S2F).

Post-CFSE-labelling, SNFT was monocultured/co-cultured for 48 h, and the proliferative index was calculated (PI_SNFT_) after normalizing by PI^day-0^ (Figure S3A). PI_SNFT_ fold-increase after NIH3T3^J1-A^_,_ NIH3T3^J1-B,^ and NIH3T3^J1-C^ co-culture were 12.32-fold, 9.34-fold, and 9.57-fold (*p* < 0.0001), respectively, with insignificant fold-difference between two intermediate clones. The NIH3T3^J1-D^ co-culture further reduced the PI_SNFT_ (3.27-fold), comparable to NIH3T3^wt^ (3.67-fold) (*p* < 0.0001) (Figure 3C). Co-culture with A2780 (4.85-fold), OAW42 (4.40-fold), and OVCAR3 (7.71-fold) (*p* < 0.001) imparted an incremental effect on proliferation (Figure 3D). Rh-Jag1 concentration gradient also showed a similar effect (Figure S3B). Compared with basal SNFT (MFI:11.75 ± 1.52), Ki-67 was maximally expressed after co-culture with OVCAR3 (MFI:25.15 ± 2.07), followed by NIH3T3^J1-A^ (MFI:22.76 ± 2.22), showing significantly higher nuclear localization, 2.13- and 1.93-fold (*p* < 0.01), respectively. OAW42-SNFT co-culture resulted in a 1.6-fold (*p* < 0.01) increase in nuclear expression (MFI:18.20 ± 1.57). NIH3T3^wt^ induction was comparable to SNFT (MFI:12.39 ± 2.21, *p* < 0.01) (Figure 3E,F).

Cancer cells also modulate invasive potential as well as proliferation for tumor sustenance [14]. SNFT, already metastatic, showed invasion 24 h post-culture (10.33 ± 0.33 cells/field). In comparison, NIH3T3^J1-A^-induction augmented invasiveness by 6.83-fold (*p* < 0.001). Similarly, OVCAR3, OAW42, and NIH3T3^J1-D^ cells induced 5.64-fold (*p* < 0.001), 2.96-fold (*p* < 0.01), and 2.09-fold (*p* < 0.05) higher invasiveness in SNFT respectively, while NIH3T3^wt^ induced non-significant invasion (1.22-fold, *p* = ns) (Figure 3G,H). Rh-Jag1 peptide treatment (25 µM) slightly increased the invasiveness (2.3-fold, *p* < 0.05) (Figure S3).

In several malignancies, Notch3 abets drug resistance [15]. CFSE-labelled SNFT was sorted after co-culturing and tested for cisplatin sensitivity. The IC_50_ of cisplatin for SNFT increased ~2–3-fold after culturing with either NIH3T3^J1-A^ (8.8 µg/mL, *p* < 0.01) or OVCAR3 (8.3 µg/mL, *p* < 0.01) compared to basal SNFT (2.7 µg/mL) and also with NIH3T3^wt^-culture (3.8 µg/mL) (Figure 3I,J). The peptide (25 µM) also imparts cisplatin resistance in SNFT (IC_50_: 6.61 µg/mL) (Figure S3C).

### 3.4. Cancer-Associated Fibroblasts (CAF) Induce Notch3 Activation in the Co-Culture Model

**Figure 4:**
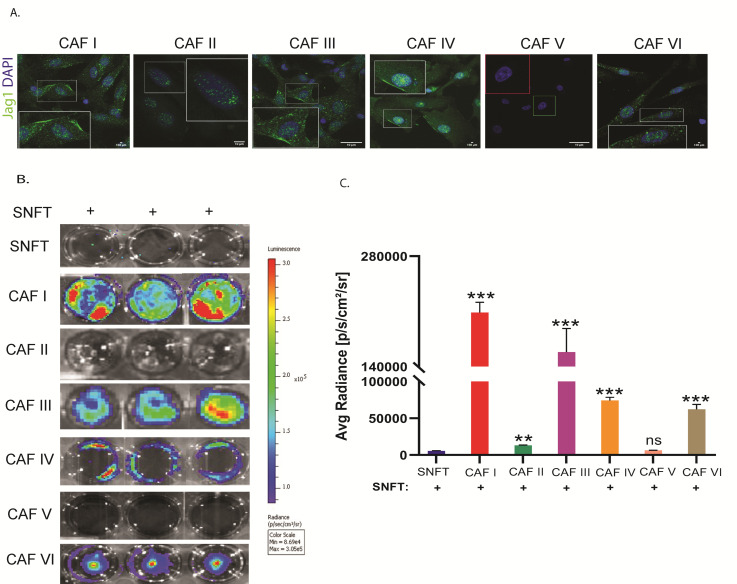
Cancer-associated fibroblasts induce Notch3 activation in the co-culture model. (**A**) Immunofluorescence images depicting the localization pattern (both membranous and cytoplasmic) of Jag1 in CAFs. CAFs from patients II and V did not show membranous Jagged-1. Notably, Jag1 expression showed heterogeneity across different cells within an individual and across patients (*n* = 1). (**B**,**C**) Co-culture of these CAFs induced differential levels of Notch3 activation in SNFT as indicated by the luciferase signal (*n* = 1). Note that CAF II and CAF V co-culture did not activate Notch3 in SNFT (*n* = 1). ** *p* ≤ 0.01, *** *p* ≤ 0.001.

We isolated CD90+ CAFs from malignant ascites of six HGSOC patients, evaluated Jagged1 status, and validated the sensor model in cancerous conditions. For four cases (CAF I, III, IV, and VI), CD90^+^ CAFs showed membranous and cytoplasmic Jagged1 expression but at differential levels. CAF-II showed low and cytoplasmic expression, and CAF-V did not have any detectable expression (Figure 4C). Differential level of Notch3 sensor activation in SNFT (CAF I:37-fold, *p* < 0.001; CAF III:28.1-fold, *p* < 0.001; CAF IV:13.2-fold, *p* < 0.001; and CAF VI:6.8-fold, *p* < 0.001 increase over SNFT-alone) was observed when Jagged1^+ve^ CAF-co-culturing (Figure 4C,D).

### 3.5. CDKN1A and VEGFA Are Two Key Differential Genes (DGs) in SNFT Post Homotypic/Heterotypic Activation of the Notch3 Pathway

**Figure 5:**
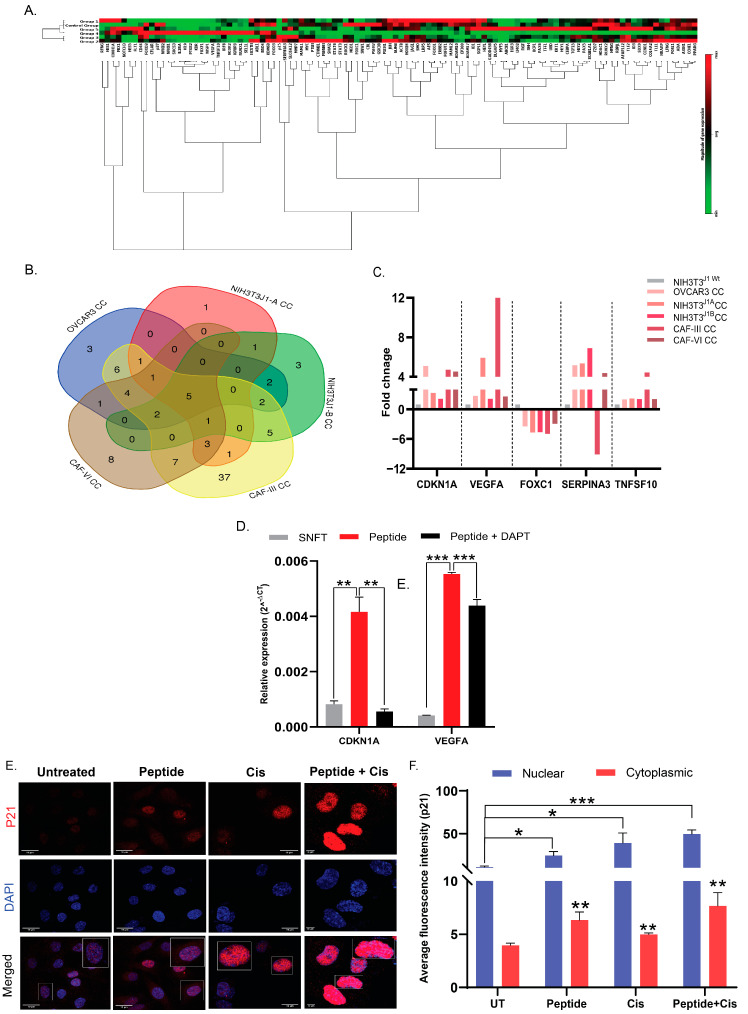
CDKN1A and VEGFA are two key differential genes (DGs) in SNFT post homotypic/heterotypic activation of the Notch3 pathway. (**A**) Modulation in 107 Notch3 target genes in FACS sorted labelled SNFT cells from co-culture (CC) with five Jagged-1 expressing cells (OVCAR3, NIH3T3^J1-A^, NIH3T3^J1-B^, CAF-III, and CAF-VI) as compared to NIH3T3^wt^ cells is represented by the heatmap. (**B**,**C**) The Venn diagram shows the unique and overlapping differential genes across five co-cultures. Among them, CDKN1A, VEGFA, and TNFSF10 exhibited significant up-regulation. At the same time, SERPINA3 (only in CAF-III co-culture SNFT) and FOXC1 showed down-regulated expression. (**D**) Expressions of CDKN1A and VEGFA (the two most significant DGs) were increased after Jag1 peptide treatment, which declined after DAPT treatment in SNFT (*n* = 2) (see Table S2 for expression values). (**E**,**F**) Immunofluorescence images showing the expression level and localization of p21 in SNFT after peptide treatment or cisplatin treatment or treatment with both. Peptide treatment significantly augmented nuclear and cytoplasmic localization of p21, which further increased after cisplatin along with peptide treatment. Cisplatin alone did not cause any change in nuclear or cytoplasmic p21 compared to untreated. The white-outlined insets showed magnified view of the areas highlighted (*n* = 3). The nuclear-cytoplasmic intensity tool, an ImageJ macros toolset, was used to quantify the expression. * *p* ≤ 0.05, ** *p* ≤ 0.01, *** *p* ≤ 0.001.

The incremental ligand expression induces an amplified activation of Notch3 through NICD-cleavage. To delineate the amplification’s summative effect on classical/non-classical target genes’ expression associated with various diseases, we performed the transcriptional profiling of SNFT across co-culture (NIH3T3^wt^, NIH3T3^J1-A^, NIH3T3^J1-B^, CAF-III, CAF-VI, and OVCAR3). Altogether normalized expression of 107 putative candidate genes was plotted (Figure 5A). The core downstream effector genes of Notch3, e.g., *pbx1, hes1*, showed increased expression indicating an activated Notch3 pathway. Across populations, five DGs (CDKN1A, VEGFA, FOXC1, TNFSF10, and SERPINA3) had a significant fold-change compared with NIH3T3^wt^ (Figure 5B). SERPINA3 (except for CAF-III-co-culture) and FOXC1 were upregulated and downregulated, respectively (Figure 5C). NICD3 causes transcriptional de-repression at the CSL-ternary complex by replacing HDAC with HAT [16]. Hence, the upregulated DGs represent a surrogate of Notch3 activation. Three genes, i.e., CDKN1A (2.15- to 5.1-fold), VEGFA (2.15- to 12.12-fold), and TNFSF10 (2.03- to 4.44-fold), were upregulated post-co-culture in SNFT (Figure 5C). Two maximally modulated genes (CDKN1A and VEGFA) were further validated (Figure 5D).

Rh-Jag1 treatment (25 µM) increased both *CDKN1A* and *VEGFA* expressions in SNFT by 5.12-fold (*p* < 0.01) and 13.75-fold (*p* < 0.0001), and DAPT attenuated this effect by 7.45-fold (*p* < 0.01) and 1.27-fold (*p* < 0.01), respectively, indicating Notch pathway-mediated regulation during co-culture (Figure 5D).

Jagged1 peptide (25 µM) resulted in a pan-cellular redistribution of p21 and the expression increased by 2.35-fold (*p* < 0.01) (nucleus) and 1.62-fold (*p* < 0.01) (cytoplasm). Cisplatin (25 µg/mL) increased nuclear localization (3.57-fold, *p* = 0.058), along with a slight increase in the cytoplasm (1.26-fold, *p* < 0.01). Intriguingly, a combinatorial treatment (peptide-cisplatin) resulted in maximum nuclear (4.53-fold, *p* < 0.001) and cytoplasm (1.94-fold, *p* < 0.01) expression of p21 in SNFT (Figure 5E,F).

### 3.6. VEGFA Regulates CSC-Non CSC Turnover in A2780^LR^ Cells, and Its Expression along with CDK2N1A Correlates with Notch3 in Metastatic HGSOC Tumors

**Figure 6:**
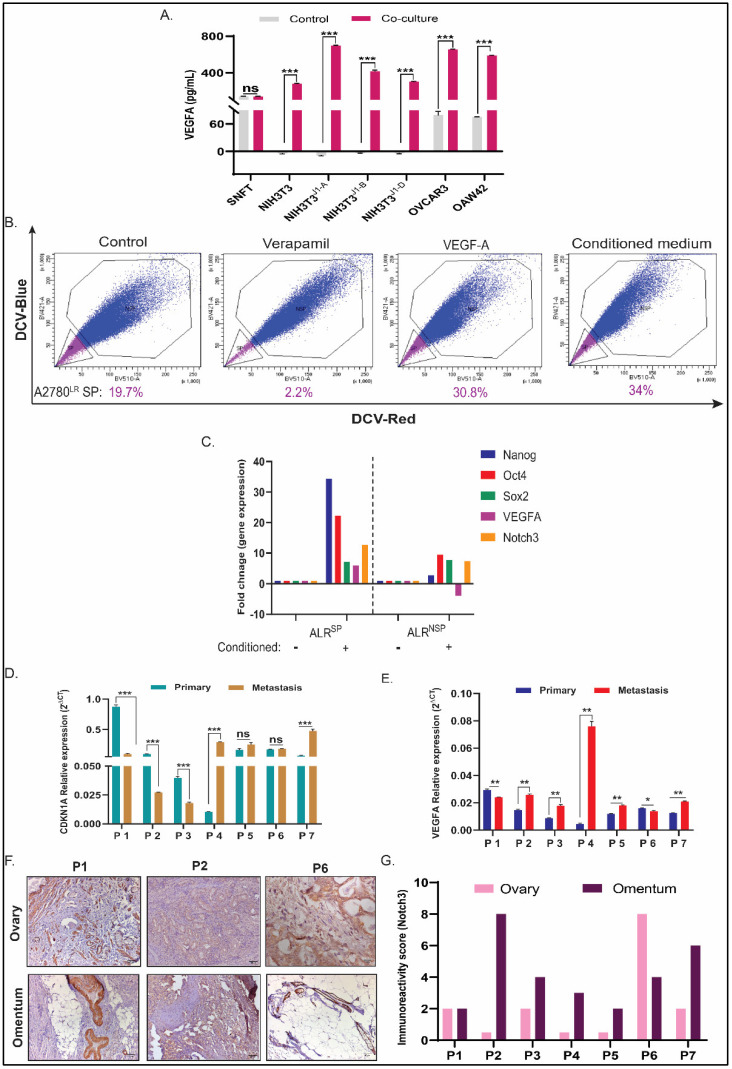
VEGFA regulates CSC to non-CSC turnover in A2780^LR^ cells, and its expression along with CDK2N1A correlates with Notch3 in metastatic HGSOC tumours. (**A**) Bar diagram shows differences in VEGFA secretion by SNFT cells after various co-culture conditions. NIH3T3J1-A induced the greatest fold-increase of VEGFA activity (~5-fold) that gradually diminishes with OVCAR3, OAW42, NIH3T3^J1-B^, NIH3T3^J1-D^, and NIH3T3^wt^ cells. (**B**) Increased side population was observed in A2780^LR^ cells after treatment of either VEGFA peptide or conditioning with a co-cultured medium containing VEGFA (*n* = 2). (**C**) The fold change for the pluripotent genes, VEGFA and Notch3, was determined after conditioning the SP and NSP fractions of A2780^LR^ cells that showed a prominent enrichment of stem-like property in SP cells compared to NSP along with a predominantly active Notch3/VEGFA axis only in SP cells. The ‘+’ sign and ‘−‘sign signified whether the cells were conditioned with the co-cultured medium or were unconditioned, respectively. (**D**,**E**) The gene expression for CDKN1A and VEGFA was compared between primary and metastatic tumors from seven paired cases of HGSOC, wherein VEGFA showed significant increase post metastasis in five cases and a decrease or no change in one case each. For CDKN1A, the expression had either increased or decreased in three patients, each with one case having no difference in expression. (**F**,**G**) Across the ovary and omentum, Notch3 showed membranous-cytoplasmic expression. The majority of the cases had higher Notch3 immunoreactive score (IRS) in metastatic tumors and no change or decrease in staining in one case each. Smooth muscle cells of blood vessels are considered an internal positive control. * *p* ≤ 0.05, ** *p* ≤ 0.01, *** *p* ≤ 0.001, ns-statistically non-significant.

SNFT secreted higher VEGFA after co-culturing with NIH3T3^wt^ (2-fold, *p* < 0.001), NIH3T3^J1-A^ (5.01-fold, *p* < 0.001), NIH3T3^J1-B^ (2.98-fold, *p* < 0.001), and NIH3T3^J1-D^ (2.16-fold, *p* < 0.001) to SNFT culture alone (Figure 6A). Both OVCAR3 and OAW42 cells had secreted VEGFA. For homotypic co-culture conditions, normalization for the feeder layer confirmed increased VEGFA secretion after OVCAR3 (4.14-fold, *p* < 0.001) and OAW42 (3.67-fold, *p* < 0.001) co-culture.

Earlier works reported that VEGFA could drive cancer-initiating stem cells in triple-negative breast cancer and non-small cell lung carcinoma, primarily through the myc/stat3/sox2 axis [17,18]. Notch3 is a known critical regulator of cancer stem cells (CSC) in ovarian carcinoma [15]. Hence, to determine whether VEGFA secretion induced by co-culture affects side population fraction, a surrogate marker for CSC characterization, DCV efflux assay, was performed on A2780^LR^ cells. Owing to its terminally resistant nature against cisplatin, A2780^LR^ showed a 19.7% SP fraction at the basal level, which increased after purified VEGFA treatment (2 ng/mL) for 7 days by 1.6-fold to 30.8%. Conditioning with co-cultured media after incubating SNFT cells upon NIH3T3^J1-A^ feeder layer for 1 week increased the SP fraction further (34%) by 1.72-fold (Figure 6A). Oct4 expression (MFI) in A2780^LR^ was also found to be increased (2.39-fold) after conditioning with the co-cultured medium. However, the percentage increase of positive cells was insignificant (1.75 in untreated v/s 2.13 in a conditioned medium treated (Figure S4A–C).

Next, we sought to determine whether the Notch3/VEGFA axis exerts differential effects upon the SP & NSP fractions in terms of their differentiation, drug resistance, and pluripotent characteristics. All these attributes are decisive for EOC progression. An equal number of sorted SP (19.3%) and NSP (70%) cells of A2780^LR^ were incubated in the supernatant of the SNFT/NIH3T3^J1-A^ co-cultured medium for a week and again sorted into SP and NSP fractions. Both differentiation of SP (SP: 39.2%; Figure S5) and minimal de-differentiation of NSP (SP: 9.3%; Figure S5) were observed (data not shown). Intriguingly, expression of pluripotent genes, e.g., *nanog*, *oct4*, and *sox2*, increased in SP and NSP factions post-conditioning compared to the untreated SP or NSP, respectively. However, the fold increase of these genes was significantly higher in conditioned-SP (*nanog*: 34.5-fold; *oct4*: 22-fold) than that of conditioned-NSP (*nanog*: 2.8-fold; *oct4*: 9.5-fold) except for *sox2* (conditioned-SP: 7.2-fold; conditioned-NSP: 7.7-fold). Also, Notch3, one of the well-known drivers of stemness, increased by 12.7-fold and 7.4-fold in SP and NSP, respectively, after conditioning. This observation has strengthened the fact that the SP population is indeed enriched with stem-like cells. Interestingly, the expression of *vegfa* increased by 6-fold in SP but plummeted by 3.9-fold in NSP cells, indicating the absence of an active Notch3/VEGFA axis in NSP cells. Due to the complicated experimental design and a small number of SP cell collections, we could not measure the secreted VEGFA independently from SP and NSP fractions after conditioning.

Finally, to assess our findings in HGSOC patient population, we measured *CDKN1A* and *VEGFA* transcripts levels from primary and corresponding metastatic tumor tissues. Among the seven patients, omental metastasis, compared to respective primary tumors, showed decreased expression of *CDKN1A* (P1, P2, P3; *p* < 0.001) in three cases and increased *CDKN1A* in another three (P4, P7; *p* < 0.001; P5, *p* = ns) with one case having no difference in expression. However, five patients showed increased *VEGFA* gene expression in metastatic tumors compared with their primary, which also showed enhanced NICD3 expression as determined by IHC. In the remaining two cases, the *VEGFA* level was reduced in the paired metastatic counterparts (P1, *p* < 0.01; P6, *p* < 0.05). The cleaved Notch3 level in these cases was not altered (P1) or decreased (P6) (Figure 6F,G). Notably, we did not observe any nuclear localization of cleaved Notch3 and this was thereby scored based upon its cytoplasmic-membranous expression (Figure S4D).

## 4. Discussion

Both ascitic and tumor tissue microenvironments in EOC, known for their complex biology during tumor initiation and progression, comprise various acellular/cellular components with a critical role in the evolution of malignancy [1]. Omentum is the most common site of distant metastasis in advanced-stage EOC, comprising 70% of all cases wherein binary interactions between either stromal/tumor or tumor/tumor cell pairs are pathologically critical [1,19]. To assess and estimate the impact of cell–cell communications (either homotypic or heterotypic), we investigated the juxtacrine interaction between CAFs/tumor cells in the context of Notch3 signaling and also whether potential heterogeneity in the CAFs or tumor cells leads to differential levels of Jagged1 ligand that can drive the Notch3 activation differentially in EOC cells and its functional consequence. Developing a one-of-its-kind luciferase-based multimeric reporter sensor in SKOV3 cells (SNFT) and co-culturing SNFT with various cell types, we investigated the effect of differential activation of Notch3 in real-time. The sensor is found to be sensitive enough to distinguish different ligand concentrations either overexpressed on the surface of NIH3T3^jag1^ clones or naturally expressing EOC cell lines and CAFs. Its sensitivity was also ascertained in the presence of a Notch-pathway inhibitor and inducer, i.e., DAPT and rh-Jag1 peptide, respectively. To the best of our knowledge, this is possibly the first-ever study to demonstrate the differential activation of the Notch pathway, particularly Notch3, by patient-derived CAFs with variable expression of Jagged1. Such differential Notch3 pathway activation elicited modulations in proliferation, invasion, and drug sensitivity of SNFT through both homo/heterotypic co-culture conditions, which were directly proportional to the level of Jagged1 expression. When compared across all the co-culture conditions, the Notch3 RT2 profiler array identified *VEGFA* and *CDKN1A* as the most upregulated candidates among the top modulated genes in SNFT. Due to Notch3 activation after co-culture, enhanced VEGFA secretion from SNFT enhanced the un-differentiated CSC population in platinum-resistant A2780 cells. Though co-culture medium did induce differentiation in pure SP cells, the pluripotent genes and Notch3 expression were enhanced several-fold higher in SP than in NSP fraction. Reduction in VEGFA expression in NSP fraction after treatment with co-culture media indicates that the Notch3/VEGFA axis is exclusively active in SP cells. Altered p21 expression/spatial regulation, consequent to Notch3 activation, was observed after rh-Jag1 peptide/cisplatin treatment. Interestingly enough, both NICD3 protein and *VEGFA* transcripts were found to be upregulated in the metastatic tumors compared to the corresponding paired primary tumors of HGSOC patients. This potential interplay of Notch3 along with p21 and VEGFA evidently emerges as critical regulators during tumor metastasis leading to the progression of ovarian carcinoma.

The potential heterogeneity in Jagged1 expression in the neighboring cells imparts differential juxtacrine activation of Notch3, rendering relevant clinical implications [20,21]. To date, six real-time monitoring strategies have been developed for Notch activation. Three of them were developed in order to study canonical Notch signaling, such as NICD/CSL luciferase complementation, *hes1*/*hes5* promoter-driven fluorescent genes, and CSL-binding sites-driven fluorescent reporters [11,22,23,24]. Despite having its own merits, each of these relied primarily upon the co-expression of a specific Notch receptor with autocrine induction of the reporter or induction by non-specific chemical inducers (EDTA). Except for the luciferase complementation, all other studies evaluated the Notch reporters in cell fate determination during CNS development. Also, there are three reported methods based on synthetically engineered Notch receptor (synNQ) approaches. The ligand-interacting EGF-like domain of the N-terminus of the Notch receptor can be replaced, e.g., with a GFP-binding nanobody. Alternatively, the downstream transcriptional effector molecule, i.e., ICD in the C-terminus, can be swapped with other transcription factors (e.g., QF) without affecting upstream cleavage events (Ref.). Also, a synthetic system has been developed by introducing different binding partners of ICD without affecting the receptor itself [25,26]. Since our focus was to investigate the functioning of intact Notch3 receptor and ligand, the SNFT sensor does not fit for comparison with synthetic Notch receptor. Our unique co-culture-based Notch reporter model utilizes the multimerized CSL-binding sites-driven firefly luciferase (which possesses a better signal-to-noise ratio), recording only ‘canonical’ signaling (ICD/CSL-mediated) and not any non-CSL mediated Notch signaling.

In contrast, any putative non-CSL-mediated form of Notch signaling should go undetected [27]. However, epithelial ovarian cancer is not known for the presence of any non-canonical form of Notch signaling; rather, the canonical Notch3 pathway is one of the major pathways responsible for promoting EOC. EOC cell lines possess different expression/levels of activation of all Notch receptors; therefore, selecting a candidate to delineate the Notch receptor-specific effect is critical, and SKOV3 is deemed appropriate for the model due to its autocrine activation of every Notch receptor except Notch3 [13]. The endogenous Notch receptors of SNFT cells and Jagged1-expressing cells (natural and overexpressed) function as the receiver and the donor. SNFT showed no significant luciferase signal owing to the absence of basal Notch3 activity, indicating non-response to NICD of other Notch receptors. The mechanism, though it requires elucidation, suggests the specificity of our system.

Similarly, the lack of induction in luciferase signal by the NIH3T3^jag1-nodder^ or TOV21G (no membrane localization of Jag1) indicates that the system’s specificity interaction between the receiver and heterogeneous levels of donor led to differential Notch3 activation, which led to downstream molecular and biological heterogeneity. The rh-Jag1 peptide or DAPT inhibitor reliably demonstrated the sensitivity of the sensor. NIH3T3^J1-A^ co-culture induced the sensor at 12 h post-culture, reached maxima at 24 h then decreased until 48 h, thus exhibiting the temporal activation kinetics. Several naturally Jagged1-expressing EOC cell lines (A2780, OVCAR3, and OAW42) were used as the feeder layer, mimicking tumor cells’ homotypic interaction. The concomitant and proportional cleavage of NICD3 across conditions deemed the model a bona fide sensor for the Notch3 pathway activity.

As a result of its pleiotropic nature, deregulation of Notch leads to aberrant effects on cellular proliferation, migration, angiogenesis, and survival [28,29,30]. In melanoma, Notch3 had been linked to invasion-migration properties of the cells [31]. Notch3 is also implicated in inhibiting cell proliferation and invasion in breast cancer through PTEN transactivation [32]. Both Ki-67 expression and CFSE-assay ascertained the hyperproliferation of SNFT by Notch3. We observed that NIH3T3^J1-A^ and OVCAR3 imparted the highest proliferative, invasive, and chemoresistance potential. However, OVCAR3 co-culture caused the maximum nuclear localization of Ki-67. Interestingly, NIH3T3^wt^ co-culture imparted marginal proliferative and invasive leverage, possibly by the PGE2 secretion, activating ERK1/2 in SNFT [33]. SKOV3 cells (Platinum-IC_50_: ~3 µg/mL) acquired further resistance post-activation of the pathway through peptide/co-culture [34]. Thus, this model can reliably measure the activation dynamics of Notch3 signaling.

Both canonical and non-canonical Notch signaling are known for initiating a transcriptional cascade that involves both the activation and the repression of target genes. The canonical Notch signaling follows a multi-tiered signaling cascade which gets strictly trans-activated (normal condition) or trans/cis-activated (malignant condition) by several ligands of delta (DLL1, DLL3, DLL4) and serrate (Jag1, Jag2) families leading to sequential cleavages and release of NICD, which translocates to the nucleus causing transcriptional modulation by co-activation complex formation [35,36]. The non-canonical Notch pathway, on the other hand, primarily comprises a CSL-independent transcriptional role or complete absence or replacement of canonical ligand for activation, which is reported in the context of several tumors [10,11,12,13]. However, epithelial ovarian cancer is not known for the presence of any non-canonical form of Notch signaling; rather, canonical Notch3 pathway is a major pathway responsible for promoting EOC. To appraise the effects of differential Notch3 activation on cellular properties, we adopted a stringent and elaborate FACS sorting strategy to isolate labelled SNFT cells after homotypic (A2780, OAW42, and OVCAR3 cells) or heterotypic (Jagged1 expressing NIH3T3 clones/patient-derived CAFs) co-cultures. A real-time profiler array was carried out on these sorted populations that identified five commonly modulated targets of Notch3 (TNFSF10, FOXC1, VEGFA, CDKN1A & SERPINA3). Perchet et al. have demonstrated that in the context of the type-I lymphoid immune function, TNFSF10, along with Stat5/IL2 pathway, co-cluster with the Notch pathway genes, though the exact mechanism of this association is not known [37]. FOXC1 is reported to be an oncogene in several cancers like basal-like breast cancer, hepatocellular carcinoma, endometrial cancer, and lymphoma [38]. In our analysis, FOXC1 was downregulated post Notch3 activation, but the exact mechanism of this observation needs further elucidation. FOXC1 protein was present in 84% of serous ovarian cystadenomas and 66.7% of borderline cystadenomas, whereas its expression was observed in only 37.5% of adenocarcinomas, which suggests that FOXC1 is a better prognosis marker [39]. SERPINA3 is commonly associated with the transition of benign tumors to invasive melanoma [40]. Except for CAF-III co-culture, we observed upregulation of this oncogene in all the conditions.

Two significantly upregulated genes, CDKN1A and VEGFA, unlike basic helix-loop-helix (bHLH) domain (*hes1*) or homeobox family (*pbx1*) members, are non-classical Notch3 targets. VEGFA is known for promoting angiogenesis [18,41]. VEGFA upregulation and secretion by SNFT cells through co-culture/peptide-induction signified the role of the Notch3 pathway in disease progression in EOC. CAF-III, inducing maximal VEGFA expression in SNFT, is particularly important in highlighting the strength of this system in delineating the molecular angiogenic cues. Interestingly, VEGFA shows a lesser DAPT-driven inhibition of peptide induction than p21. VEGFA expression is known to be regulated by Notch3/Jag1 axis through the zeb1 transcription factor [42]. Thus, feedback mechanisms other than Notch3/Jag1/zeb1 axis possibly contribute to this reduced VEGFA inhibition. Bevacizumab, a VEGF inhibitor, is currently in use as maintenance therapy for advanced-stage HGSOC due to the well-established role of VEGFA in EOC angiogenesis. Recently, in primary ovarian carcinoma culture, VEGFA had been shown to promote tumor-initiating cells through Src-DNMT3A-driven miR-128-2 methylation and Bmi1 upregulation [43]. VEGFA is also known to modulate cancer stem cell-enriched side population in breast cancer patients through non-canonical PKA/β-catenin pathway and ABCG2/ABCB1 drug efflux transporters [44]. Intriguingly, the conditioned medium of co-culture of SNFT and NIH3T3^Jag1A^ cells imparted slightly higher induction of SP fraction than purified VEGF treatment in platinum-resistant A2780^LR^ cells. This effect might have resulted from other secreted factors present in the co-culture medium. When a pure fraction of the SP and NSP population were incubated with the co-culture medium, both differentiation of SP and a minimal de-differentiation (less than 10%) of NSP were observed. Such differentiation and de-differentiation phenomena were not reported earlier and could be attributed to several factors present in co-culture media. We are working towards the identification of these factors. Intriguingly, along with pluripotent genes (Oct4, SOX2, and Nanog), the Notch3/VEGFA axis is highly expressed exclusively in SP cells and probably contributes to pro-tumorigenic activities. Our data thus indicate a broader role of the Notch3/VEGFA axis in the enrichment of undifferentiated, drug-resistant CSC-like population and neo angiogenesis during EOC metastasis. Further investigation on this axis would open up the prospect of therapeutic intervention at the pre-metastatic niche formation.

p21, a G1/S checkpoint protein, binds to CDK1 and induces cellular senescence after stress-induced p53-activation [45]. However, studies have shown that p21 could play a myriad of pro-tumorigenic roles depending on its cellular localization [46]. p21 phosphorylation at Thr-145 and Ser-146, caused by AKT1, sequesters it in the cytoplasm. This imparts apoptosis-resistance by sequestering pro-caspase-3 and thus inhibiting Fas-mediated cell death [47]. Also, nuclear p21 induces proliferation through CCND1 accumulation and CDK4/6 association [48]. Notch3 can regulate p21 transcription through *hes1* [49]. Our data show prominent and expected nuclear localization of p21 after cisplatin treatment which redistributed to cytoplasm post dual treatment of cisplatin and rh-Jag1, indicating a probable anti-apoptotic fate adapted by the cells. This molecular event possibly leads to the acquirement of platinum resistance, as observed in this study. Intriguingly, rh-Jag1 treatment alone also significantly increased cytoplasmic localization of p21 which is possibly due to an increase in pAkt level [50]. Finally, we observed a biological correlation between Notch3 protein upregulation and transcriptional induction of VEGFA in five metastatic HGSOC tumors compared with their primary counterparts. Interestingly, in P6 we observed a concomitant decrease in Notch3 as well as VEGFA expression. However, the CDKN1A encoding p21 did not show any particular correlation with Notch3 [51].

Notch3 signaling is tightly regulated in a multi-tiered fashion and determines the cell fate decision during normal development and pathogenesis. Herein, we have elucidated the functional and molecular aspects of trans-regulation of Notch3 by heterogeneous cells from the malignant ascites of EOC and the possible involvement of Notch3 at various facets in disease progression. This is probably the first report of homotypic cell–cell interaction in EOC leading to Notch3 activation. Redesigning this reporter system as a platform for a high throughput library will enable the screening of Notch3-associated therapeutic candidates in the future.

## 5. Conclusions

This study conclusively established that differential ligand induction by the tumor microenvironmental cells leads to differential Notch3 activation in tumor cells in a homo- and heterotypic fashion. The differential activation of Notch3 resulted in phenotypic modulation in SKOV3 cells in a proportional manner, and p21 and VEGFA are two critical genes through which Notch3 imparts its effects during these interactions. This study uncovers an unknown role of the Notch3/VEGFA axis in EOC progression.

## Data Availability

The data is contained in the current manuscript and supplementary material.

## Supplementary Materials

The following supporting information can be downloaded at: https://www.mdpi.com/2072-6694/14/14/3365/s1, Figure S1: Characterization of wild type and mutant Jagged-1 expressing clones; Figure S2: Differential expression of Jagged-1 in EOC cells induce differential Notch3 activation in SNFT; Figure S3: (Effect of rh-Jag1 peptide on cellular proliferation, cisplatin-sensitivity and invasion. Figure S4: Enhanced OCT4 expression in co-culture and Notch3 expression in patient tumor blocks; Figure S5: The SP and NSP cells were sorted from A2780LR and separately conditioned with the co-culture medium followed by re-acquiring and re-sorting. Figure S6. Full-length blots original immunoblots used in the manuscript. Table S1: Primer list (for qPCR). Table S2: Real time data (Figure 5D) (normalized expression). Table S3: RT2 profiler array gene list (normalized expression).
