# Correction: Mukherjee et al. Homo and Heterotypic Cellular Cross-Talk in Epithelial Ovarian Cancer Impart Pro-Tumorigenic Properties through Differential Activation of the Notch3 Pathway. *Cancers* 2022, *14*, 3365

**DOI:** 10.3390/cancers16040685

**Published:** 2024-02-06

**Authors:** Souvik Mukherjee, Asmita Sakpal, Megha Mehrotra, Pratham Phadte, Bharat Rekhi, Pritha Ray

**Affiliations:** 1 Imaging Cell Signaling and Therapeutics Lab, Advanced Centre for Training Research and Education in Cancer, Navi Mumbai 410210, India; smukherjee@actrec.gov.in (S.M.); lgunjal@ymail.com (A.S.); meghameh13@gmail.com (M.M.); pratham1122@gmail.com (P.P.); 2 Homi Bhabha National Institute, BARC Training School Complex, Anushaktinagar, Mumbai 400094, India; rekhi.bharat@gmail.com; 3 Tata Memorial Hospital, Dr. E Borges Road, Parel, Mumbai 400012, India

In the original publication [1], the complete invasion assay results have taken three control wells into consideration to calculate the average (Figure 3H).

1. In the original publication, the images shown for peptide treatment in Figure 1D,I are not correct. *The peptide panel in Figure 1D was mistakenly copied from the manually scaled-up image of the 12 h SNFT-NIH3T3^J1-A^ co-culture image of Figure 2A. Similarly, the peptide panel in Figure 1I was also mistakenly copied from the manually scaled-up image of the 24 h SNFT-NIH3T3^J1-A^ co-culture image of Figure 2A*. We now have added the actual images of Figure 1D (peptide panel) and Figure 1I (peptide & DAPT+Peptide).

**Figure 1:**
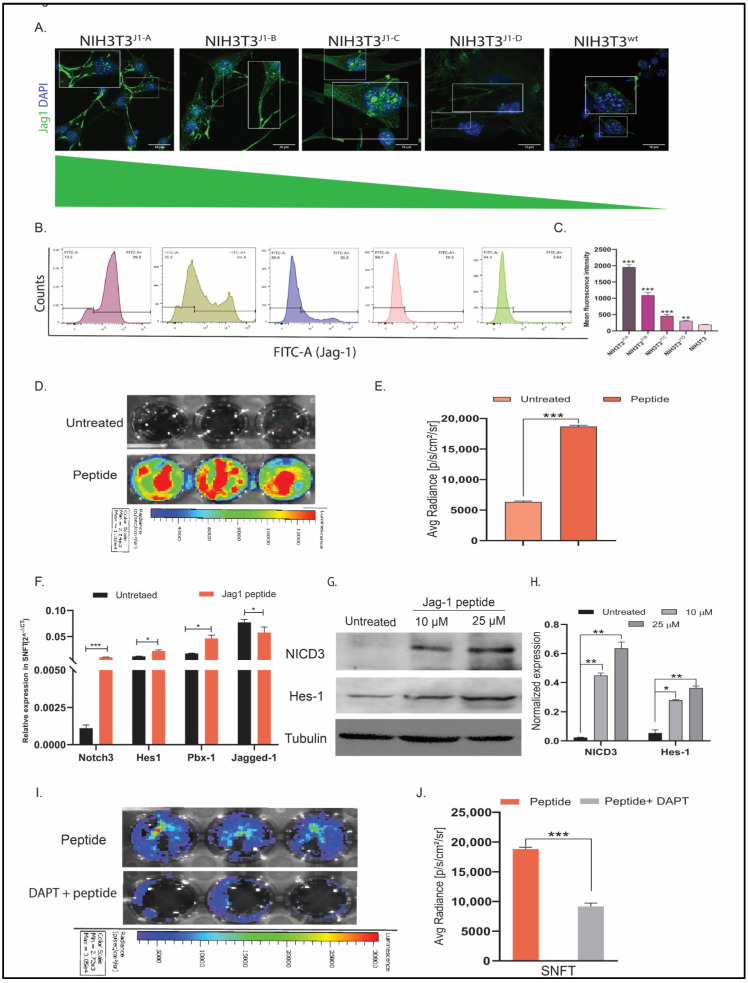
Establishment of differential NIH3T3^jag1^ clones and Notch3 reporter sensor. (**A**) Immunofluorescence images representing the membranous localization of Jag1 in NIH3T3 clones (white outlined inset images showed the magnified view of the areas highlighted). (**B**,**C**) Flow cytometry panel representing the quantitation of cell surface expression of Jag1 through mean fluorescence intensity, which indicated a differential degree of Jag1 expression across the clones (*n* = 3). (**D**,**E**) Live cell bioluminescence imaging of SNFT cells after Jag-1 peptide treatment (**D**) and graphical representation of the same (*n* = 2) (**E**) showing increased Notch3-sensor promoter activity. (**F**,**G**) Several Notch3 targets (*hes1*, *pbx1*, *notch3*) showed upregulation after peptide-induced activation at the transcript level (*n* = 3). Further, the Western blot of NICD3 and Hes1 after differential Jag1 induction in SNFT (**G**) (also see Figure S6A) and the densitometric quantification of the proteins (**H**) (housekeeping control: alpha-tubulin, *n* = 2) highlight that the NICD3 cleavage and its target protein expression increased differentially upon induction due to the pathway activation. (**I**,**J**) Activation/inhibition kinetics of live-cell bioluminescence image depicting that peptide-induced promoter activity attenuated after DAPT blockade (*n* = 2). * *p* ≤ 0.05, ** *p* ≤ 0.01, *** *p* ≤ 0.001.

2. In the original publication, there was a typographic error in *Figure 2C* as published. The label SCFT should have been SNFT.

In addition, there was a mistake in *Figure 2G* as published. Due to a fold in the original Western blot membrane, *the tubulin band of the SCFT control appeared to be from another blot*. We, therefore, replaced the whole figure with another replicate experiment.

**Figure 2:**
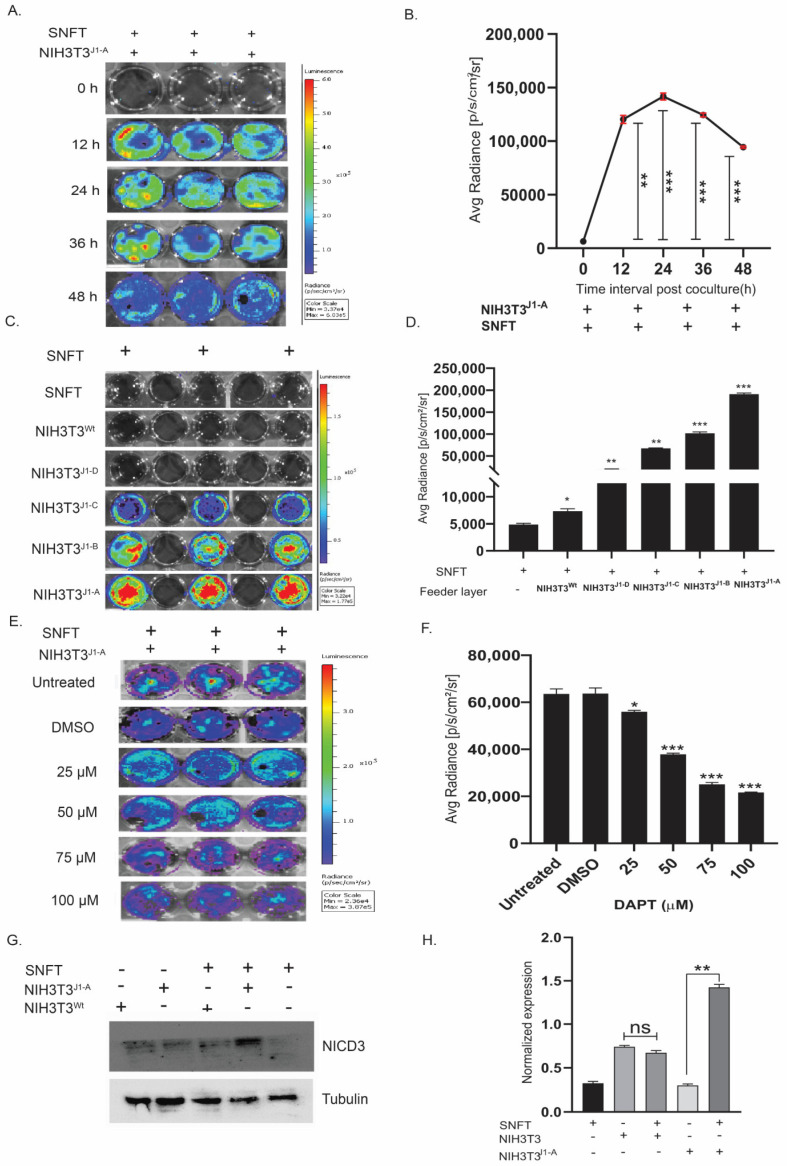
Establishment of a unique co-culture model of Notch3 activation-sensor expressing tumor cells (SNFT) and differentially expressing Jagged-1 fibroblasts (NIH3T3^jag−1^). (**A**,**B**) Temporal kinetics of Notch3 sensor activity in SNFT in co-culture with NIH3T3^J1-A^ cells showed an initial activation after 12 h of 18.9-fold (*p* < 0.01), which reaches its maxima at 24 h (22.3-fold, *p* < 0.001) and subsequently dropped by 48 h (14.8-fold, *p* < 0.001). The two-tailed error bar (red) represented the SEM (*n* = 3). (**C**,**D**) Bioluminescence images of co-culture between SNFT and different Jag1 clones of NIH3T3 (**C**) show a linearly proportional relationship between Jagged1 expression and Notch3 promoter activity as represented graphically in (**D**) (*n* = 3 for each clone). (**E**,**F**) Dose-inhibition kinetics are represented by the live-cell images (**E**) and graphically (**F**), which shows that concentration between 50 µM to 75 µM of DAPT results in a significant reduction in the NIH3T3J1-A mediated Notch3 activity in SNFT (*n* = 3). All three real-time live-cell imaging experiments were independently performed and represented by respective scale bars. (**G**,**H**) Immunoblot of sorted SNFT cells post-co-culture and graphical analysis show increased release of NICD3 in SNFT when co-cultured with NIH3T3^wt^ (not significantly) and with NIH3T3^J1-A^ (~3-fold, *p* ≤ 0.01). The ‘+’ sign and ‘−‘ sign signify the presence and absence of the cells in co-culture, respectively (*n* = 2) (also see Figure S6B). * *p* ≤ 0.05, ** *p* ≤ 0.01, *** *p* ≤ 0.001.

3. In the original publication, there was an error in *Figure 5E* as published. *The p21 (red) channel image of cisplatin treatment shows a halo around one of the cells, which should not be in that image as magnified insets are shown only for merged images. This sub-image has been corrected*.

**Figure 5:**
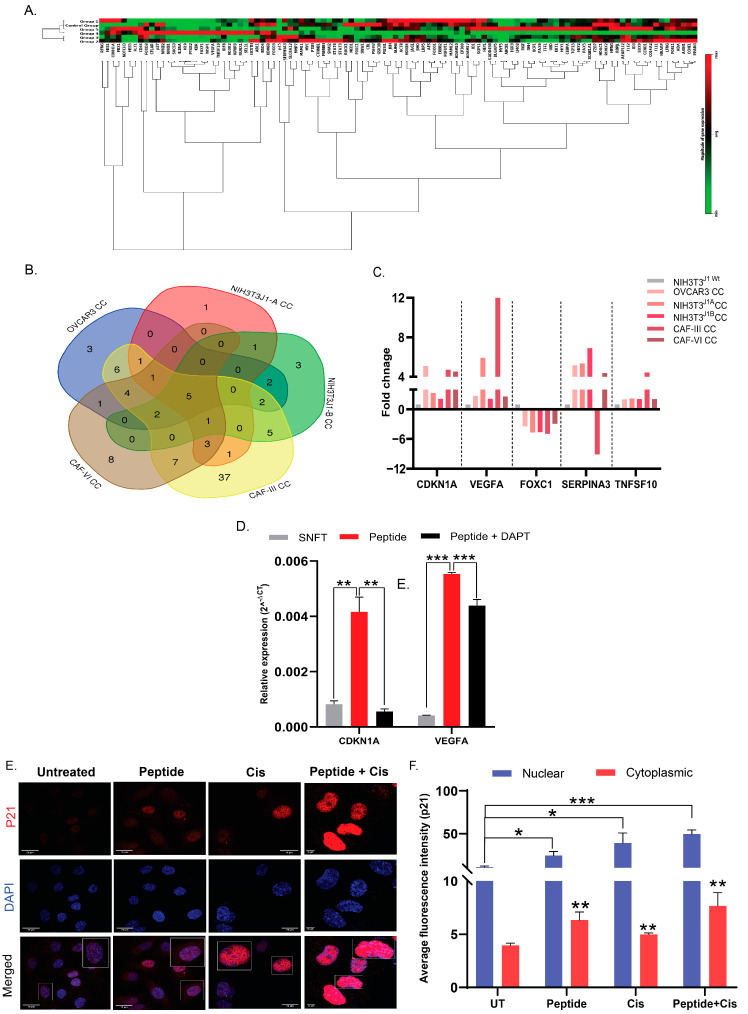
CDKN1A and VEGFA are two key differential genes (DGs) in SNFT post homotypic/heterotypic activation of the Notch3 pathway. (**A**) Modulation in 107 Notch3 target genes in FACS sorted labelled SNFT cells from co-culture (CC) with five Jagged-1 expressing cells (OVCAR3, NIH3T3^J1-A^, NIH3T3^J1-B^, CAF-III, and CAF-VI) as compared to NIH3T3^wt^ cells is represented by the heatmap. (**B**,**C**) The Venn diagram shows the unique and overlapping differential genes across five co-cultures. Among them, CDKN1A, VEGFA, and TNFSF10 exhibited significant up-regulation. At the same time, SERPINA3 (only in CAF-III co-culture SNFT) and FOXC1 showed down-regulated expression. (**D**) Expressions of CDKN1A and VEGFA (the two most significant DGs) were increased after Jag1 peptide treatment, which declined after DAPT treatment in SNFT (*n* = 2) (see Table S2 for expression values). (**E**,**F**) Immunofluorescence images showing the expression level and localization of p21 in SNFT after peptide treatment or cisplatin treatment or treatment with both. Peptide treatment significantly augmented nuclear and cytoplasmic localization of p21, which further increased after cisplatin along with peptide treatment. Cisplatin alone did not cause any change in nuclear or cytoplasmic p21 compared to untreated. The white-outlined insets showed magnified view of the areas highlighted (*n* = 3). The nuclear-cytoplasmic intensity tool, an ImageJ macros toolset, was used to quantify the expression. * *p ≤* 0.05, ** *p ≤* 0.01, *** *p ≤* 0.001.

4. In the original publication, there was a typo in *Figure 6F and Figure S4D* as published. *The scale bars were written as ‘10 cm, which should be 10 μm*. The corrected *Figures of the IHC panels* appear below.

**Figure 6:**
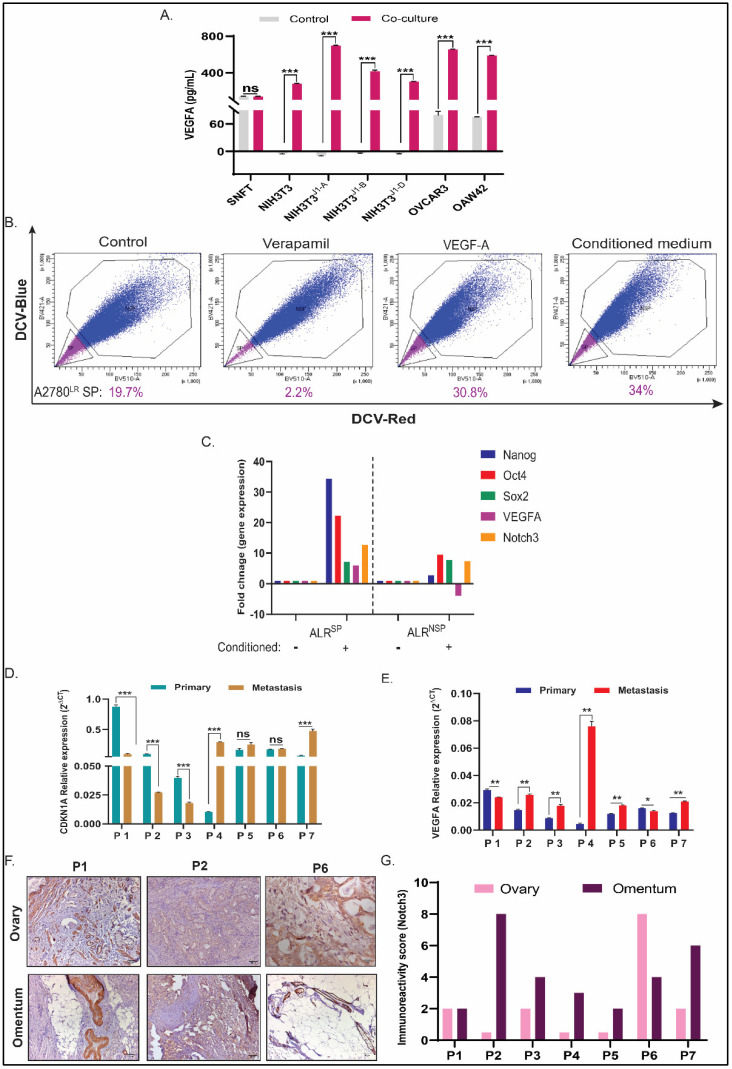
VEGFA regulates CSC to non-CSC turnover in A2780^LR^ cells, and its expression along with CDK2N1A correlates with Notch3 in metastatic HGSOC tumours. (**A**) Bar diagram shows differences in VEGFA secretion by SNFT cells after various co-culture conditions. NIH3T3^J1-A^ induced the greatest fold-increase of VEGFA activity (~5-fold) that gradually diminishes with OVCAR3, OAW42, NIH3T3^J1-B^, NIH3T3^J1-D^, and NIH3T3^wt^ cells. (**B**) Increased side population was observed in A2780^LR^ cells after treatment of either VEGFA peptide or conditioning with a co-cultured medium containing VEGFA (*n* = 2). (**C**) The fold change for the pluripotent genes, VEGFA and Notch3, was determined after conditioning the SP and NSP fractions of A2780^LR^ cells that showed a prominent enrichment of stem-like property in SP cells compared to NSP along with a predominantly active Notch3/VEGFA axis only in SP cells. The ‘+’ sign and ‘−’ sign signified whether the cells were conditioned with the co-cultured medium or were unconditioned, respectively. (**D**,**E**) The gene expression for CDKN1A and VEGFA was compared between primary and metastatic tumors from seven paired cases of HGSOC, wherein VEGFA showed significant increase post metastasis in five cases and a decrease or no change in one case each. For CDKN1A, the expression had either increased or decreased in three patients, each with one case having no difference in expression. (**F**,**G**) Across the ovary and omentum, Notch3 showed membranous-cytoplasmic expression. The majority of the cases had higher Notch3 immunoreactive score (IRS) in metastatic tumors and no change or decrease in staining in one case each. Smooth muscle cells of blood vessels are considered an internal positive control. * *p ≤* 0.05, ** *p ≤* 0.01, *** *p ≤* 0.001, ns-statistically non-significant.

The authors sincerely apologize for all these mistakes and any inconvenience caused, and state that the scientific conclusions are unaffected. All these corrections were approved by the Academic Editor, and the original publication has also been updated

## References

[1] Mukherjee S., Sakpal A., Mehrotra M., Phadte P., Rekhi B., Ray P. (2022). Homo and Heterotypic Cellular Cross-Talk in Epithelial Ovarian Cancer Impart Pro-Tumorigenic Properties through Differential Activation of the Notch3 Pathway. Cancers.

