# Supplementary material for: Homo and Heterotypic Cellular Cross-Talk in Epithelial Ovarian Cancer Impart Pro-Tumorigenic Properties through Differential Activation of the Notch3 Pathway
Source: Cancers (Basel). 2022 Jul 11;14(14):3365. doi: 10.3390/cancers14143365 (PMC9319742; doi:10.3390/cancers14143365)
Supplement: Supplementary file 1 [file cancers-14-03365-s001.zip › cancers-1782557-Supplementary.pdf]

Supplementary Material: Homo and Heterotypic Cellular Cross-Talk in Epithelial Ovarian Cancer Impart Pro-Tumorigenic Properties through Differential Activation of the Notch3 Pathway

Souvik Mukherjee, Asmita Sakpal, Megha Mehrotra, Pratham Phadte, Bharat Rekhi and Pritha Ray

Supplementary Figure S1.

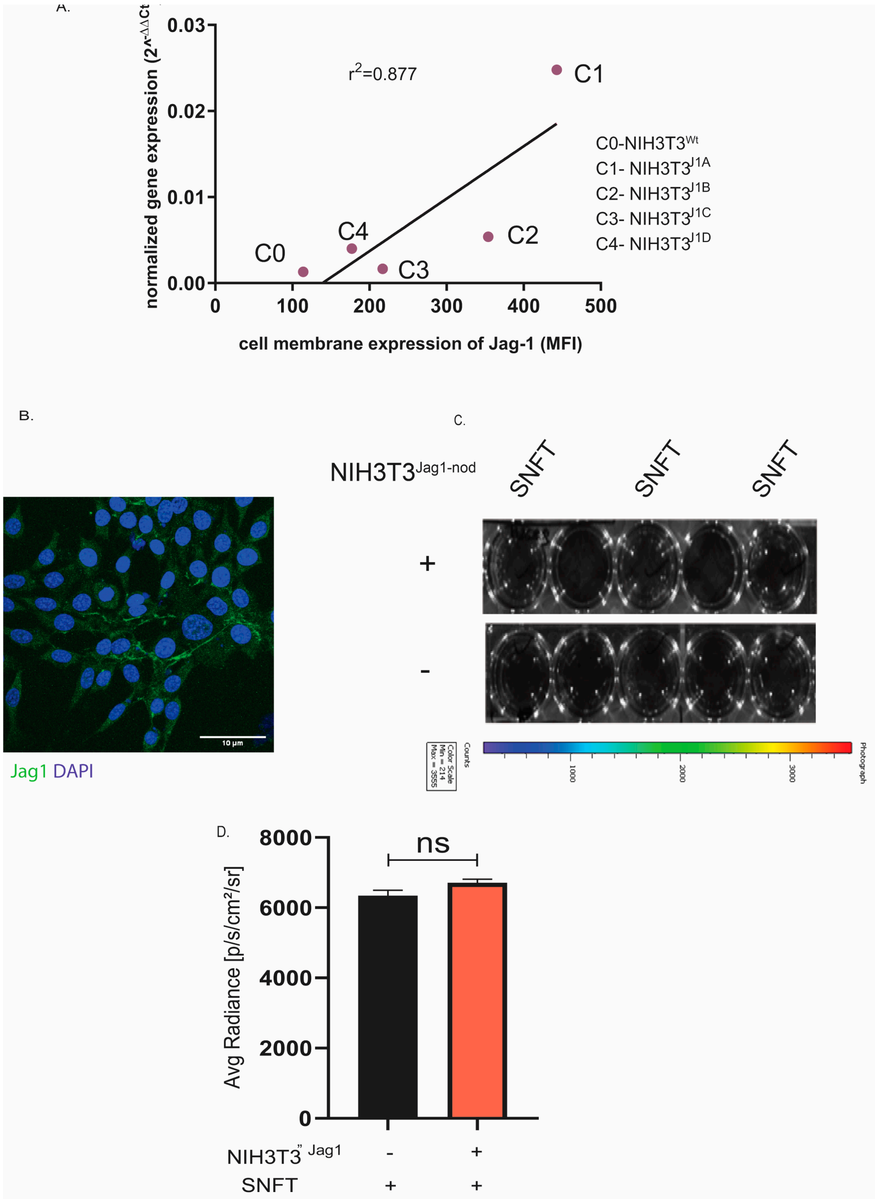

Figure S1. Characterization of wild-type and mutant Jagged-1 expressing clones. (**A**) Membrane expression of Jag1 was calculated as MFI and compared with the normalized transcript levels across all the NIH3T3jag1 overexpressing clones which showed a good positive correlation. (**B**) The mutant Jag1 localizes on the plasma membrane but (**C**,**D**) did not result in any Notch3 activation, as evident from the absence of luciferase activity.

Supplementary Figure S2.

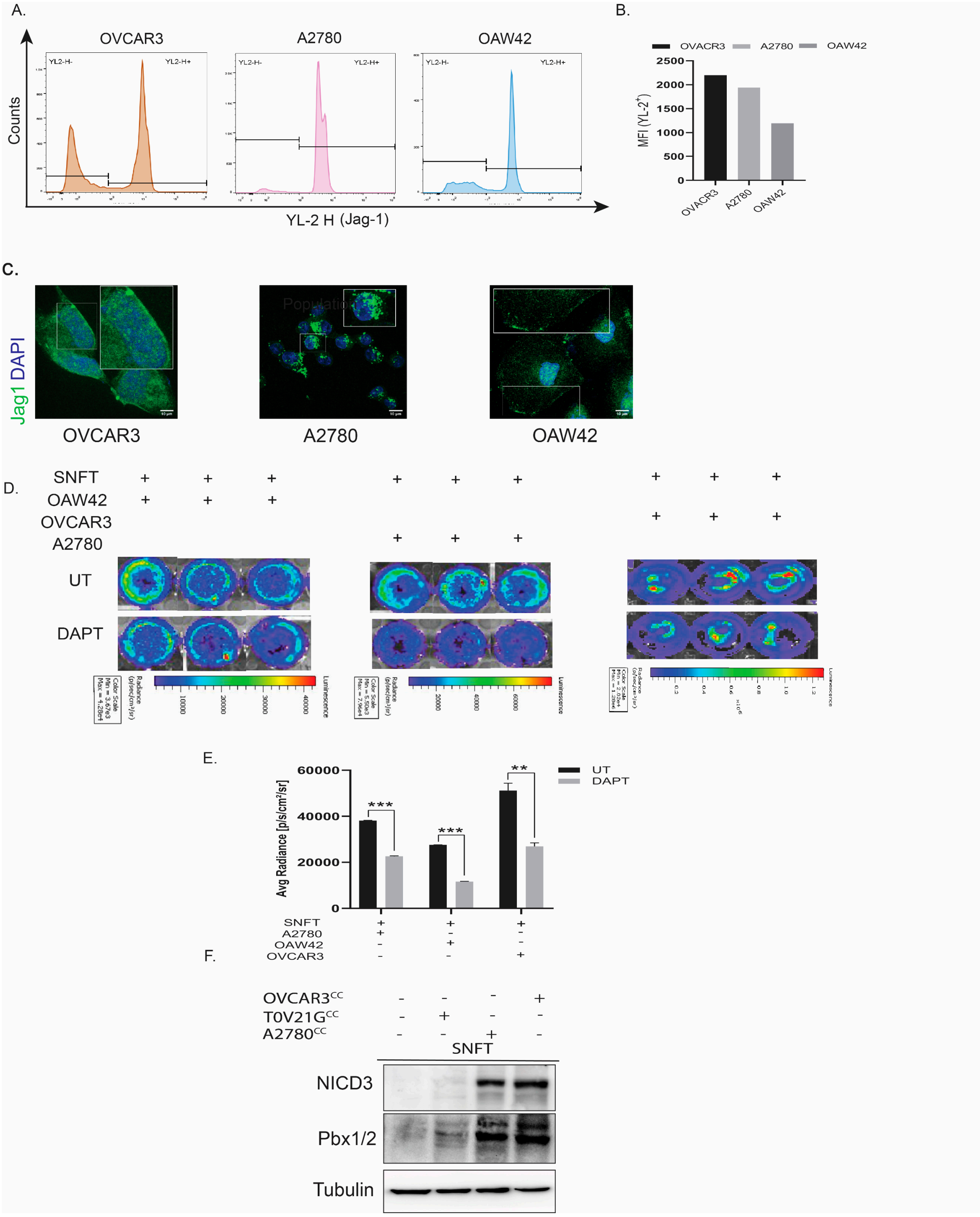

Figure S2. Differential expression of Jagged-1 in EOC cells induces differential Notch3 activation in SNFT. (**A**,**B**) The levels of Jag1 were measured by flow cytometry across EOC cell lines naturally expressing ligand, which shows a differential pattern in the following descending order: OVCAR3, A2780, and OAW42. (**C**) The membrane localization of Jag1 was confirmed by immunofluorescence in all the cell lines. (**D**,**E**) Upon co-culturing with SNFT, these cell lines induced differential activation of Notch3, which was sensitive to DAPT inhibition. (**F**) The expression of NICD3 and Pbx1/2, a Notch3 target protein, upon co-culture with OVCAR3 and A2780 but not with TOV21G, confirmed the upstream cleavage of Notch3 which is a pre-requisite for its activity. ** $p < 0.01$, *** $p < 0.001$.

Supplementary Figure S3.

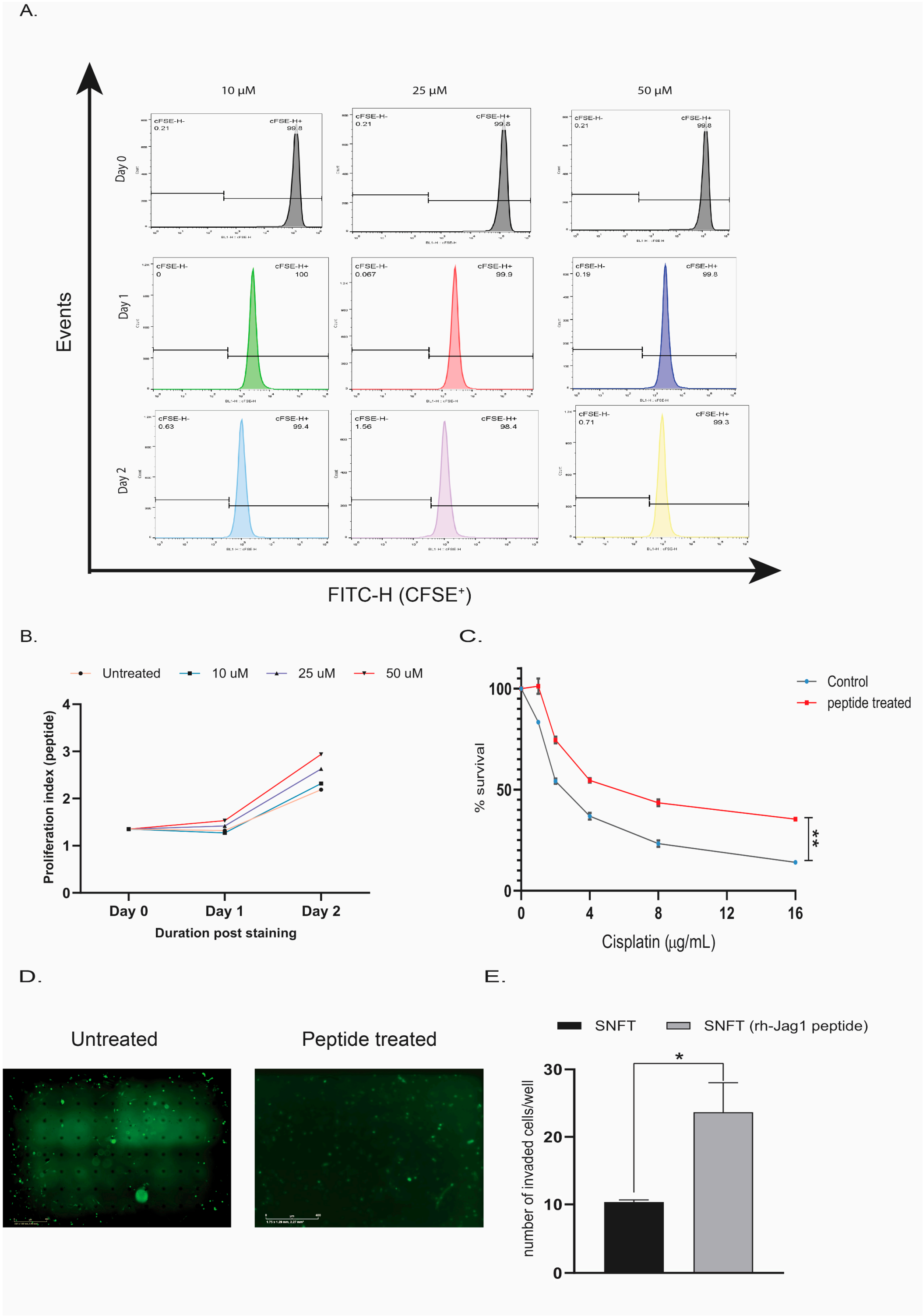

Figure S3. Effect of the rh-Jag1 peptide on cellular proliferation, cisplatin-sensitivity, and invasion. (**A**,**B**) The gradient of rh-Jag1 peptide concentration induced a proportional effect on the SNFT proliferation, as evident from the proliferation index. (**C**) The peptide treatment significantly reduced the cisplatin sensitivity of SNFT, as shown by the MTT assay. (**D**,**E**) The invasion potential of SNFT was augmented by the peptide-mediated Notch3 pathway activation. * $p < 0.05$.

Supplementary Figure S4.

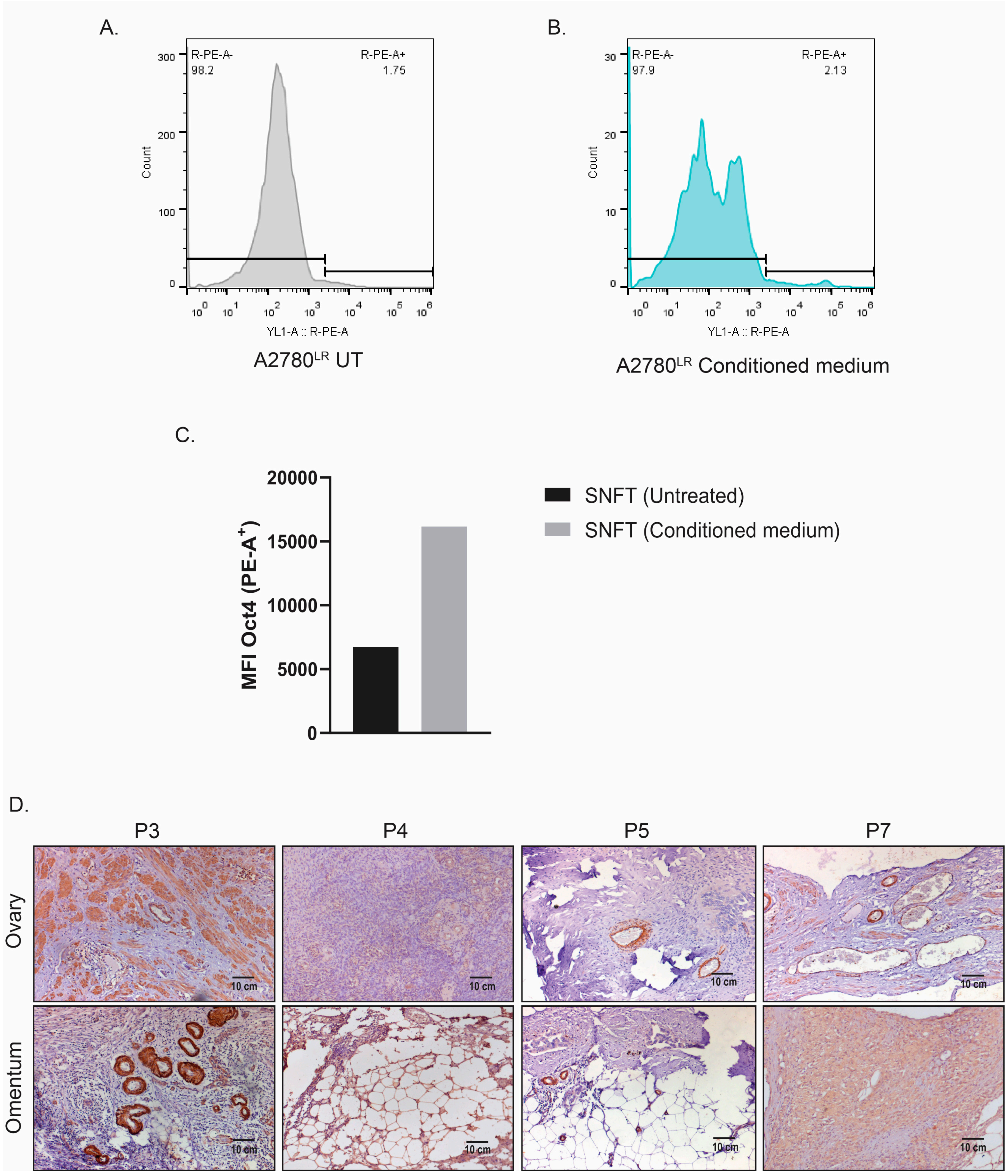

Figure S4. Enhanced OCT4 expression in co-culture and Notch3 expression in patient tumor blocks. (**A**–**C**) The percentage of Oct4 positive A2780^{LR} cells increased from 1.75% to 2.13%, and the MFI of the positive cells increased by 2.39-fold from 6748 to 16163. (**D**) Notch3 expression in paired cases of primary and metastatic tumors in HGS patients. Smooth muscle cells of blood vessels are considered as an internal positive control.

Supplementary Figure S5:

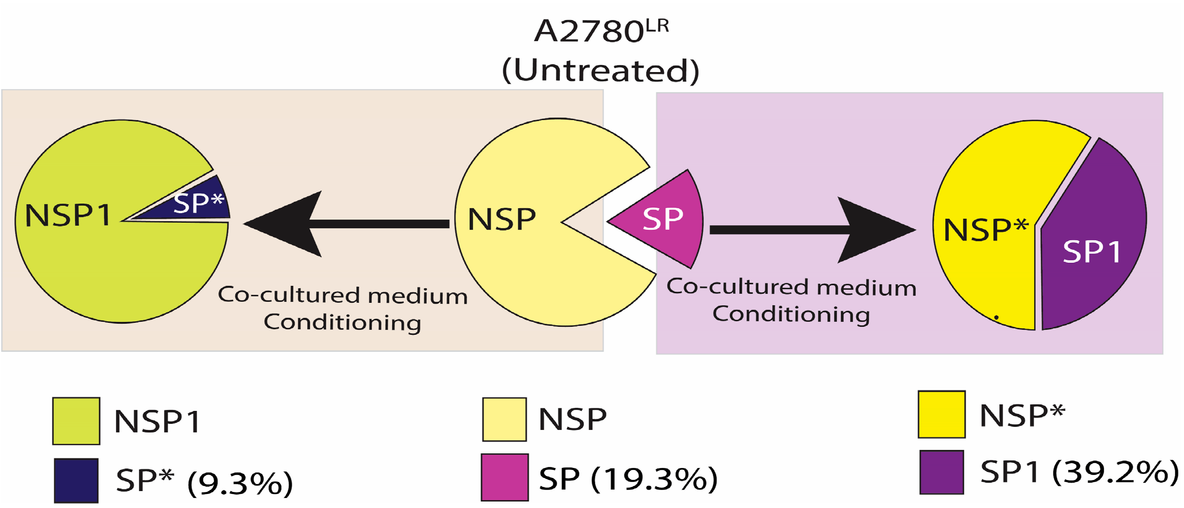

Figure S5. Schematic for SP/NSP differentiation/de-differentiation post-conditioning. The SP and NSP cells were sorted from A2780^{LR} and separately conditioned with the co-culture medium, followed by re-acquiring and re-sorting. Upon re-acquiring, the SP fraction in the previously sorted SP cells was 39.2% (SP1). In the case of NSP also, a small fraction (9.3%) of cells had de-differentiated to SP (SP*).

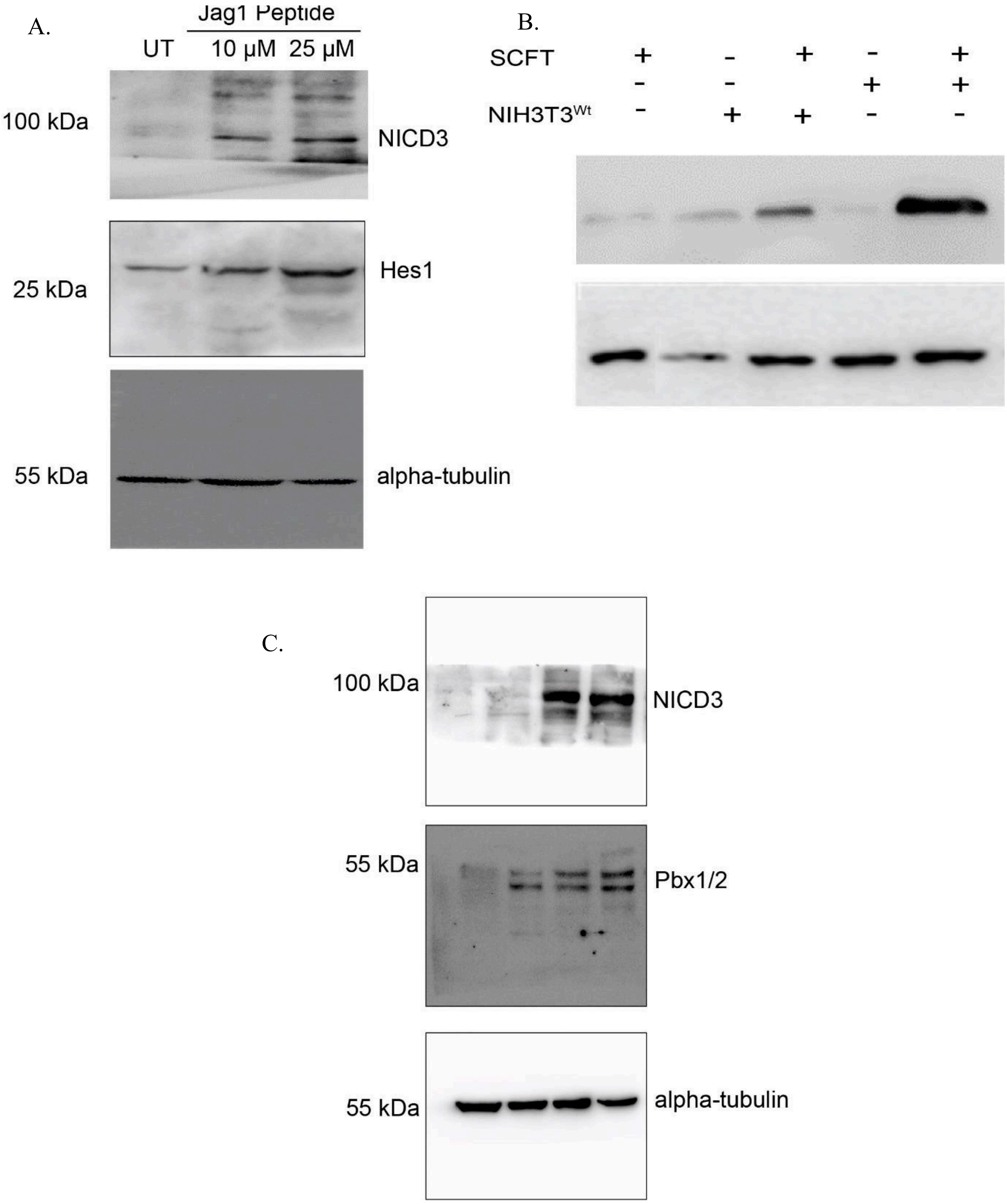

Figure S6. Full-length blots original immunoblots used in the manuscript. **A**. Original immunoblot used in Figure 1G. **B**. Original immunoblot used in Figure 2G. **C**. Original immunoblot used in supplementary figure S2.

Table S1- Primer list (for qPCR):

Gene	Oligo sequence	Product length
Notch-3	Forward: 5′-CTGTGGCCCTCATGGTATCTG-3′<br>Reverse: 5′-GCATGGGTTGGGGTCACAGTC-3′	118 bp
Jag-1	Forward: 5'-GGCAACACCTTCAACCTCAAGG-3'<br>Reverse: 5'-ACAAGCAACGTATAGGACCTCG -3'	98 bp
Hes-1	Forward: 5'-TGAAGAAAGATAGCTCGCGG-3'<br>Reverse: 5'-GGTACTTCCCCAGCACACTT -3'	138 bp
Pbx-1	Forward: 5'-AACTCGGCTGGTTCTTCCAG-3'<br>Reverse: 5'-CTGTGATTGCAACGTTGGCTC-3'	123 bp
CDKN1A	Forward: 5′-AGGTGGACCTGGAGACTCTCAG-3′<br>Reverse: 5′-TCCTCTTGGAGAAGATCAGCCG-3′	194 bp
VEGFA	Forward: 5'- GCACCCATGGCAGAAGG -3'<br>Reverse: 5′- CTCGATTGGATGGCAGTAGCT -3′	90 bp
VEGFR2	Forward: 5′- GGAACCTCACTATCCGCAGAGT-3′<br>Reverse: 5′- CCAAGTTCGTCTTTTCCTGGGC-3′	132 bp
GAPDH	Forward: 5'-TGCACCACCAACTGCTTAGC-3'<br>Reverse: 5'-GGCATGGACTGTGGTCATGAG-3'	87 bp
Nanog	Forward: 5′- CTCCAACATCCTGAACCTCAGC-3′<br>Reverse: 5′- CGTCACACCATTGCTATTCTTCG-3′	115 bp
Oct4	Forward: 5′- CCTGAAGCAGAAGAGGATCACC -3′<br>Reverse: 5′- AAAGCGGCAGATGGTCGTTTGG-3′	106 bp
Sox2	Forward: 5′- GCTACAGCATGATGCAGGACCA-3′<br>Reverse: 5′- TCTGCGAGCTGGTCATGGAGTT-3′	135 bp

Table S2- Real time data (Figure 5D) (normalized expression):

	SNFT			Peptide			Peptide +DAPT		
CDKN1A	0.000593	0.000994	0.000879	0.00501	0.0043	0.00318	0.000362	0.000649	0.000654
VEGFA	0.000433	0.000395	0.000421	0.005432	0.005523	0.005643	0.004382	0.004778	0.004019

Table S3- RT2 profiler array gene list (normalized expression):

No.	Gene Symbol	2^ (-Avg. (Delta (Ct)) (OVCAR3 coculture)	2^ (-Avg. (Delta (Ct)) (NIH3T3^{J1-A} coculture)	2^ (-Avg. (Delta (Ct)) (NIH3T3^{J1-B} coculture)	2^ (-Avg. (Delta (Ct)) (NIH3T3^{Wt} coculture)	2^ (-Avg. (Delta (Ct)) (CAF-III coculture)	2^ (-Avg. (Delta (Ct)) (CAF-VI coculture)
1	NOTCH3	0.000906	0.000510	0.000469	0.000427	0.000945	0.001443
2	JAG1	0.038750	0.025557	0.021941	0.025678	0.002842	0.026763
3	LFNG	0.000860	0.003144	0.001397	0.002374	0.000550	0.002688
4	MFNG	0.001395	0.001263	0.001100	0.001826	0.000217	0.001832
5	NUMB	0.026673	0.028921	0.017788	0.027178	0.011802	0.019888
6	RFNG	0.011693	0.009350	0.006409	0.007170	0.008064	0.006892
7	ADAM10	0.062175	0.023231	0.011257	0.049182	0.024881	0.036222
8	ADAM17	0.013234	0.007328	0.001670	0.009397	0.006352	0.010596
9	NCSTN	0.028425	0.029191	0.016044	0.023643	0.030909	0.018477
10	PSEN1	0.031615	0.040116	0.018475	0.031359	0.016108	0.027918
11	PSEN2	0.002144	0.002939	0.002342	0.004334	0.001316	0.002871
12	PSENEN	0.005499	0.011264	0.005096	0.008308	0.003652	0.006443
13	EP300	0.097272	0.049810	0.039003	0.078414	0.047134	0.045501

14	HDAC1	0.026790	0.026041	0.022824	0.032587	0.008400	0.021584
15	MAML1	0.008553	0.010438	0.006605	0.007935	0.005764	0.003713
16	MAML2	0.004532	0.003130	0.002325	0.004178	0.001930	0.002567
17	NCOR2	0.035706	0.028474	0.024529	0.045735	0.073228	0.019927
18	RBPJL	0.000821	0.000596	0.000808	0.001143	0.000304	0.001309
19	SNW1	0.005523	0.009377	0.005860	0.011738	0.004797	0.008479
20	CDKN1A	0.005592	0.003642	0.002357	0.001097	0.005184	0.004970
21	FOSL1	0.192820	0.255335	0.179120	0.204339	0.052198	0.253005
22	ID1	0.016210	0.012851	0.005256	0.011929	0.000186	0.019825
23	NFKB1	0.006605	0.010307	0.008817	0.007647	0.022367	0.010395
24	AXIN1	0.014047	0.024305	0.014039	0.021467	0.006234	0.019138
25	CTNNB1	0.013246	0.052290	0.016665	0.027952	0.007548	0.011322
26	TLE1	0.013528	0.025235	0.018646	0.023408	0.003817	0.014161
27	AES	0.054601	0.033149	0.021508	0.022685	0.009240	0.022429
28	ABL1	0.004589	0.005813	0.003297	0.005239	0.004942	0.003821
29	FOXC1	0.000004	0.000016	0.000010	0.000066	0.000013	0.000022
30	GADD45B	0.012747	0.019076	0.009101	0.021725	0.001461	0.037394
31	ID3	0.013766	0.049887	0.023599	0.023628	0.001831	0.067351
32	IGFBP3	0.379572	0.049626	0.082277	0.104560	1.151540	0.089009
33	JUN	0.096390	0.098922	0.066895	0.089559	0.066864	0.089435
34	PTGS2	0.000527	0.000333	0.000690	0.000361	0.741357	0.001422
35	SGPL1	0.020590	0.016477	0.010723	0.019528	0.010715	0.021106
36	SOCS3	0.005547	0.010038	0.005495	0.006761	0.027870	0.011249
37	VEGFA	0.042791	0.093641	0.033995	0.015811	0.191571	0.041283
38	TNFSF10	0.001542	0.001660	0.001265	0.000760	0.003370	0.001576
39	UBD	0.000808	0.000542	0.000749	0.000845	0.000370	0.001899
40	CCND1	0.107555	0.166439	0.127672	0.115253	0.026836	0.218173
41	p27	0.001043	0.002686	0.001954	0.004068	0.004233	0.002766
42	CCNE1	0.003037	0.004575	0.002903	0.003770	0.000883	0.003288
43	CD44	0.186329	0.344023	0.397777	0.262646	0.475713	0.469084
44	ERBB2	0.368815	0.179701	0.155676	0.194782	0.001693	0.144547
45	AFAP1L2	0.001530	0.005865	0.005371	0.004333	0.000362	0.006834
46	SOX9	0.002580	0.006902	0.003690	0.002762	0.000445	0.007692
47	PAX6	0.000315	0.000154	0.000227	0.000262	0.000099	0.000500
48	MYF5	0.000288	0.000291	0.000584	0.000326	0.000190	0.085882
49	SNAI2	0.037435	0.135123	0.045939	0.102087	0.040254	0.069387
50	HES1	0.048624	0.032409	0.032408	0.021694	0.040172	0.029229
51	HES5	0.000014	0.000011	0.000014	0.000029	0.000107	0.000248
52	HEY1	0.000615	0.000538	0.000634	0.000601	0.000186	0.001134
53	HEY2	0.000504	0.000439	0.000699	0.000514	0.000216	0.000861
54	HEYL	0.000184	0.000075	0.000140	0.000096	0.000029	0.000206
55	NFKB2	0.009287	0.020349	0.013147	0.022412	0.023995	0.013948
56	PPARG	0.007503	0.012919	0.007182	0.012112	0.000352	0.007366
57	STAT6	0.022172	0.042308	0.011280	0.017243	0.005965	0.023455
58	RUNX1	0.014588	0.016393	0.009724	0.015321	0.032820	0.012761
59	PBX1	0.001857	0.001430	0.001321	0.000864	0.001641	0.001887
60	SLC6A12	0.000167	0.000199	0.000256	0.000165	0.000121	0.000244
61	TCF15	0.000068	0.000082	0.000099	0.000075	0.000031	0.000132
62	RUNX2	0.018624	0.014810	0.013705	0.027563	0.003001	0.015426
63	SERPINA3	0.005362	0.005543	0.007155	0.001037	0.000111	0.004538

64	LRP5	0.002124	0.001606	0.001601	0.001281	0.000614	0.000988
65	FIGF	0.000003	0.000346	0.000761	0.000729	0.000260	0.001502
66	PAX5	0.000691	0.000412	0.000908	0.000710	0.000218	0.001265
67	POFUT1	0.006694	0.003711	0.003166	0.004690	0.001298	0.002377
68	EGR3	0.001393	0.004836	0.002994	0.000984	0.002089	0.009906
69	p15	0.000392	102938.347338	102938.989503	0.000005	0.000664	0.000136
70	FOXD3	0.000079	0.000106	0.000157	0.000160	0.000155	0.000120
71	FOXF1	0.000117	0.000178	0.000464	0.000354	0.002153	0.000652
72	ACTB	3.749541	3.264261	1.961611	3.171487	1.601949	2.776234
73	GAPDH	1.000000	1.000000	1.000000	1.000000	1.000000	1.000000
74	DLGAP5	0.002080	0.003448	0.001846	0.005520	0.000339	0.012088
75	ABCB1	0.000314	0.000690	0.000439	0.000415	0.000427	0.003228
76	MYC	0.037738	0.087695	0.049488	0.052821	0.010816	0.000700
77	COL4A2	0.051372	0.072927	0.044263	0.053124	0.011313	0.091181
78	FLT1	0.000402	0.000381	0.000704	0.000467	0.026079	0.033022
79	KDR	0.001370	0.001680	0.001446	0.001514	0.008746	0.001522
80	TFF1	0.000914	0.000389	0.000833	0.000702	0.000374	0.001451
81	TLE2	0.000080	0.000128	0.000130	0.000116	0.000030	0.000141
82	SCGB1A1	0.000624	0.000395	0.000786	0.000734	0.000226	0.001208
83	NRARP	0.000079	0.000267	0.000204	0.000178	0.000014	0.000142
84	ADA	0.004914	0.006744	0.004526	0.004840	0.001778	0.005477
85	FZD7	0.004710	0.005178	0.005310	0.006831	0.001385	0.005176
86	SMO	0.007673	0.006245	0.005083	0.005317	0.000816	0.005561
87	WISP1	0.001039	0.000537	0.001156	0.000822	0.006157	0.001947
88	SEL1L	0.025983	0.021874	0.016047	0.022832	0.080284	0.014274
89	GSK3B	0.021325	0.024323	0.020266	0.032979	0.012134	0.022458
90	MMP7	0.000776	0.000918	0.001455	0.000646	0.000234	0.000945
91	LMO2	0.000570	0.000820	0.000745	0.000557	0.000359	0.001331
92	HOXB4	0.006040	0.004530	0.004713	0.005763	0.000289	0.003973
93	KRT1	0.000607	0.000343	0.000688	0.000534	0.000269	0.001692
94	SHH	0.000304	0.000225	0.000499	0.000380	0.000239	0.000764
95	CBL	0.011755	0.016004	0.010900	0.027214	0.005904	0.009869
96	H19	0.000360	0.000680	0.000866	0.000594	0.013896	0.001090
97	CFLAR	0.006369	0.012374	0.008326	0.011688	0.018154	0.007960
98	IL2RA	0.000678	0.000746	0.000751	0.000632	0.005689	0.001161
99	STAT3	0.056306	0.077614	0.040212	0.047086	0.039286	0.041168
100	ROCK1	0.011743	0.020533	0.018117	0.024450	0.011215	0.010027
101	FOXO1	0.001680	0.001276	0.000671	0.000840	0.000295	0.000638
102	CEBPA	0.000744	0.000557	0.000657	0.000568	0.000258	0.001436
103	UCP1	0.000404	0.000343	0.000625	0.000698	0.000349	0.001241
104	IRF8	0.000861	0.000524	0.001073	0.000646	0.002155	0.000003
105	KDM6B	0.010962	0.024422	0.016594	0.020437	0.024067	0.000003
106	PTEN	0.002740	0.005264	0.002816	0.003245	0.001745	0.000003
107	BRD4	0.065447	0.052899	0.039689	0.100076	0.094157	0.000003
108	MED1	0.008767	0.010840	0.007062	0.016664	0.001749	0.000003
109	FOXO3	0.028588	0.051572	0.025145	0.059350	0.041172	0.000003
